# Antidepressant-like Activity of a Triple Combination of Venlafaxine, Codeine and Nor-Binaltorphimine in the Mouse Tail Suspension Test

**DOI:** 10.3390/ijms27219719

**Published:** 2026-10-29

**Authors:** Cristina Lungu, Călin Muntean, Horia Păunescu, Ruxandra-Cristina Marin, Florin-Adrian Lungu, Mihnea Costescu, Cristina Isabel Viorica Ghiță, Ion Fulga, Oana Andreia Coman

**Affiliations:** 1 Department No. 14 of Orthopedics, Anesthesia and Intensive Care, Faculty of Medicine, “Carol Davila” University of Medicine and Pharmacy, 050474 Bucharest, Romania; cristina.lungu@umfcd.ro; 2 Department III Functional Sciences, Discipline of Medical Informatics and Biostatistics, “Victor Babeș” University of Medicine and Pharmacy, 300041 Timișoara, Romania; cmuntean@umft.ro; 3 Department of Pharmacology, Clinical Pharmacology and Pharmacotherapy, Faculty of Medicine, “Carol Davila” University of Medicine and Pharmacy, 050474 Bucharest, Romania; mihnea.costescu@umfcd.ro (M.C.); isabel.ghita@umfcd.ro (C.I.V.G.); ion.fulga@umfcd.ro (I.F.); oana.coman@umfcd.ro (O.A.C.); 4 Doctoral School, “Carol Davila” University of Medicine and Pharmacy, 020021 Bucharest, Romania; florin-adrian.lungu@drd.umfcd.ro

**Keywords:** venlafaxine, codeine, nor-binaltorphimine, κ-opioid receptor, µ-opioid receptor, tail suspension test, multi-target pharmacology, treatment-resistant depression, molecular docking, serotonin transporter

## Abstract

About one-third of patients with major depressive disorder do not remit on conventional monoaminergic antidepressants. Venlafaxine (VEN) is a classic antidepressant that inhibits serotonin and norepinephrine reuptake. Venlafaxine does not bind opioid receptors, yet its antidepressant-like action requires intact µ-opioid signalling. The dynorphin/κ-opioid axis exerts an opposing, dysphoria-promoting influence. A possible question is whether combining serotonin and noradrenaline reuptake inhibition, µ-opioid receptor activation by codeine (COD) and sustained κ-opioid receptor blockade produces an antidepressant-like effect larger than that of the components given alone. Male NMRI mice were tested in the forced swim test (FST) (one experiment) and in the tail suspension test (TST) (three experiments) using a parallel-design (n = 10 to 17 per group; 277 animals), as follows: VEN 10 to 40 mg/kg in both tests—FST and TST; COD 10 to 40 mg/kg added to VEN 20 mg/kg in TST; and nor-binaltorphimine (norBNI) 10 mg/kg, given 24 h before, combined with VEN 20 mg/kg and COD 20 mg/kg and compared with norBNI alone, COD alone and norBNI + VEN, with amitriptyline (AMI) 20 mg/kg as the positive control in the TST. The three agents and two reference ligands were also docked into eight experimental human structures of the monoamine transporters and of the μ, κ and δ opioid receptors. VEN did not reduce FST immobility at any dose but produced a dose-dependent reduction in the TST, reaching −66.2% at 40 mg/kg. COD did not potentiate VEN 20 mg/kg at any of three COD doses. The triple combination, in the TST, reduced immobility by 65.7% relative to saline (Dunnett *p* = 0.013), an effect of similar magnitude to AMI with no significant difference detected between them, whereas norBNI alone, COD alone and norBNI + VEN did not reach significance. The design was underpowered for intermediate effects (the calculated n should be about 36 per group, unapproved because of ethical considerations). The triple combination was not significantly more effective than norBNI + VEN (*p* = 0.375), so the contribution of codeine remains undetermined. Docking reproduced the experimental pose of all eight co-crystallised ligands and was consistent with VEN occupying the transporter S1 site, COD the μ-opioid pocket and norBNI the κ receptor. A triple serotonin-adrenergic and opioid combination produced an antidepressant-like effect that none of the single treatments tested in the same cohort reached, but the fixed-dose design does not establish cooperativity between all three mechanisms. The docking results are consistent with, but do not prove, engagement of three separate sites.

## 1. Introduction

Major depressive disorder (MDD) is among the most prevalent and disabling psychiatric conditions worldwide: approximately 332 million people live with a depressive disorder [1], and depressive disorders rank among the leading contributors to years lived with disability [2], a burden projected decades ago [3]. Beyond persistent low mood, anhedonia and suicidal ideation, MDD is clinically heterogeneous in its treatment response, and cognitive dysfunction is now recognised as a principal determinant of functional impairment that persists in a substantial proportion of patients after symptomatic remission [4,5].

First-line pharmacotherapy still relies on monoaminergic agents, the selective serotonin reuptake inhibitors (SSRIs) and the serotonin and noradrenaline reuptake inhibitors (SNRIs). Venlafaxine (VEN) is a non-tricyclic bicyclic phenylethylamine SNRI whose transporter selectivity is markedly serotonin-biased in vitro, with a human serotonin transporter K_i_ of about 82 nM against about 2480 nM at the noradrenaline transporter [6]. This asymmetry translates into a dose-dependent mechanism: at low doses, VEN behaves predominantly as a serotonin reuptake inhibitor, whereas at higher doses, noradrenaline reuptake inhibition is progressively recruited, a pattern documented in rodent models [7] and in patients across the 75 to 375 mg/day range [8,9]. Critically, for the present work, the original biochemical characterisation of the compound reported no measurable affinity for opioid receptors [10].

The behavioural consequences of this dose dependence are directly relevant to immobility-based screening. In mice, VEN increased spontaneous locomotor activity at 16–64 mg/kg [7], although the literature is not uniform: noradrenaline transporter blockade has also been reported to reduce locomotion in a novel environment [11], and no locomotor change accompanied the antidepressant-like effect in other studies [12]. Because the forced swim test (FST) and tail suspension test (TST) score behavioural suppression as their primary outcome, any drug-induced shift in motor activation can confound interpretation [13,14], and this confound is most likely precisely in the dose range where VEN recruits noradrenergic transmission.

Despite established clinical efficacy, roughly one-third of patients with MDD fail to achieve remission. In the STAR*D cohort, the cumulative remission rate across four sequential treatment steps reached 67%, leaving about one-third of patients unremitted [15], and consensus statements and claims-based estimates agree that at least 30% of patients meet the criteria for treatment-resistant depression (TRD) [16,17,18]. Together with the delayed onset of clinically meaningful response [19], although early separation from placebo is detectable within the first week [20], this has motivated interest in strategies capable of engaging monoaminergic and opioid signalling simultaneously.

The three compounds studied here were selected because their structures mirror complementary pharmacological profiles. The bicyclic phenylethylamine architecture of VEN and its dual monoamine reuptake inhibition have long been noted to resemble those of tramadol, despite the absence of direct opioid receptor agonism [21]. Codeine (COD) is the 3-O-methyl ether of morphine: substitution of the phenolic hydroxyl reduces opioid receptor affinity by roughly an order of magnitude while leaving the µ/δ and µ/κ selectivity ratios essentially unchanged, so potency is lost, but the pharmacophore is preserved [22]. Nor-binaltorphimine (norBNI) is a bivalent morphinan dimer, the first highly selective κ-opioid receptor antagonist described [23,24].

Although VEN does not bind opioid receptors directly, its antidepressant-like effect depends on an intact opioid system. In the mouse FST, this effect is antagonised by the non-selective antagonist naloxone, whereas β-funaltrexamine, naloxonazine, naltrindole and norBNI, selective for µ_1_/µ_2_, δ and κ receptors, respectively, are each individually ineffective, which indicates that no single receptor subtype accounts for the interaction [25]. More directly, the antidepressant-like effect of VEN is abolished in µ-opioid receptor knockout mice without any accompanying change in locomotor activity [12]. Convergent evidence comes from nociception, where the antinociceptive action of VEN in acute rodent models is opioid-sensitive, particularly in combination regimens [26,27,28]; the two behavioural domains nonetheless dissociate pharmacologically, since norBNI and naltrindole attenuate VEN antinociception [26] but not its antidepressant-like effect [25], so opioid involvement in VEN pharmacology is domain-specific rather than global. The broader hypothesis that opioid dysregulation contributes to treatment resistance has been articulated in detail elsewhere [29,30].

The dynorphin/κ-opioid receptor system exerts an opposing influence. κ-Receptor activation produces dysphoria and psychotomimesis in humans [31] and increases FST immobility in rodents, an effect reversed by κ antagonists [32]. κ signalling encodes the dysphoric component of stress [33] and mediates stress-induced aversive states within limbic and amygdala’s circuits [34,35,36], while presynaptic κ receptors on mesolimbic dopamine terminals suppress dopamine release, a restraint that κ blockade lifts [37]. The link to dopamine is not a simple linear chain, since κ-induced aversion requires p38 MAPK signalling in ventral tegmental dopamine neurons rather than inhibition of dopamine release as such [38].

norBNI is remarkable for the duration of its action: a single administration produces κ blockade lasting from two to more than four weeks [39,40], a non-competitive persistence attributable to c-Jun N-terminal kinase 1 activation rather than to sustained receptor occupancy [41,42]. Central norBNI reduces FST immobility and upregulates hippocampal brain-derived neurotrophic factor (BDNF) mRNA, although both effects were detected on the first day only [43], and κ antagonism prolongs the antidepressant-like effect of ketamine in stress-exposed mice well beyond the pharmacokinetic window of either agent, an effect that neither drug alone maintained [44]. Such durable adjunctive efficacy is the principal rationale for including κ blockade in a multi-target combination.

Clinical development has reinforced this direction while exposing its limits. Buprenorphine/samidorphan showed adjunctive efficacy in inadequate responders [45], although subsequent phase 3 trials gave mixed results [46]. The selective κ antagonist aticaprant improved ventral striatal reward responsiveness in the FAST-MAS proof-of-mechanism trial [47,48], yet κ antagonist monotherapy and augmentation trials have so far produced modest or inconclusive results [49,50], and prototypical antagonists such as norBNI are themselves poor clinical candidates because their persistent pharmacodynamic effects complicate safety testing and trial design [51,52,53]. This contrast between mechanistic promise and monotherapy underperformance is what motivates testing κ blockade as a component of a combination rather than as a standalone treatment.

Opioid and monoaminergic crosstalk has been examined most directly with COD. In the mouse TST, COD produces a naloxone-reversible antidepressant-like effect, and sub-effective doses of COD combined with the SSRIs fluoxetine or citalopram markedly reduce immobility, whereas COD combined with the noradrenaline reuptake inhibitor desipramine or with the SNRI duloxetine produces no such potentiation, which indicates that the interaction is selectively serotonergic [54]; behavioural analysis of the test further separates opioid-type from monoaminergic response patterns [55]. Whether these interactions extend to VEN, a serotonergic-preferring SNRI at low to intermediate doses [6,7], and whether they are modified by concurrent κ blockade has, to our knowledge, not been tested systematically.

Whether these three mechanisms can act together depends on how each compound distributes across its molecular targets. VEN inhibits the human serotonin transporter with a Ki of 82 nM and the noradrenaline transporter with a Ki of 2480 nM, which is why it is serotonin-biased at low doses and progressively dual at higher ones [6,7], and it has no measurable affinity for opioid, alpha-adrenergic, histaminergic, muscarinic or dopamine D2 sites [10], so every opioid and dopaminergic consequence of VEN must be indirect. Two indirect routes are relevant here: in the prefrontal cortex, extracellular dopamine is cleared largely by the noradrenaline transporter, so noradrenaline reuptake blockade raises prefrontal dopamine without any action at the dopamine transporter itself [56]; and in the nucleus accumbens, κ blockade lifts the presynaptic restraint on dopamine release [37]. COD supplies a third input, since μ receptors on local GABAergic interneurons disinhibit ventral tegmental dopamine neurons [57]. The combination therefore rests on a structural premise, namely that each agent occupies the target set attributed to it and does not occupy the others. Behavioural end points cannot test that premise, which is why the present study includes a computational part.

The FST and TST remain the most extensively validated behavioural assays for antidepressant screening [58,59]. The FST was introduced by Porsolt and colleagues [60,61] and inspired the murine TST [62]. Immobility is now interpreted as an adaptive stress-coping strategy rather than an analogue of human depressive symptomatology [63,64], and the assays retain their place because of their sensitivity to clinically active antidepressants and their reproducibility across laboratories [58,65]. The two paradigms differ in motor demand, thermoregulatory load and pharmacological sensitivity: the murine TST is generally more responsive to serotonergic and dual-action compounds, whereas the FST shows greater strain-dependence and susceptibility to locomotor confounding [66,67], so using both yields complementary information.

We therefore designed a four-step study to test the hypothesis that simultaneous engagement of complementary monoaminergic and opioid mechanisms, namely SNRI-mediated monoaminergic enhancement by VEN, μ-opioid activation by COD and sustained κ blockade by norBNI, generates an antidepressant-like response greater than that produced by the components individually. We characterised VEN across the FST and TST, asked whether COD potentiates VEN and evaluated the triple combination against its monotherapies and binary combinations. In a fifth computational step, we docked the three compounds, together with AMI and morphine as reference ligands, into the orthosteric sites of SERT, NET, DAT and the μ, κ and δ opioid receptors in order to establish whether the target assignment underlying the combination is structurally supported. Throughout this paper, cooperativity is used in a strictly operational sense, meaning that a statistically detectable effect is present when the mechanisms are combined and absent when the components are given alone in the same cohort. Used in this way, the term is weaker than potentiation, which requires that one agent without pharmacological effect increase the effect of another, and weaker than synergy, which requires a combined effect exceeding the isobolographic prediction of additivity; it does not exclude simple additivity and it does not by itself establish that every component contributes. The assays used here are acute screening paradigms in naive animals: they model neither chronic stress nor prior failure of an antidepressant, so treatment-resistant depression enters this study as the clinical motivation for a multi-target design and not as a property of the model.

## 2. Results

### 2.1. Venlafaxine Does Not Reduce Immobility in the Forced Swim Test

**Figure 1:**
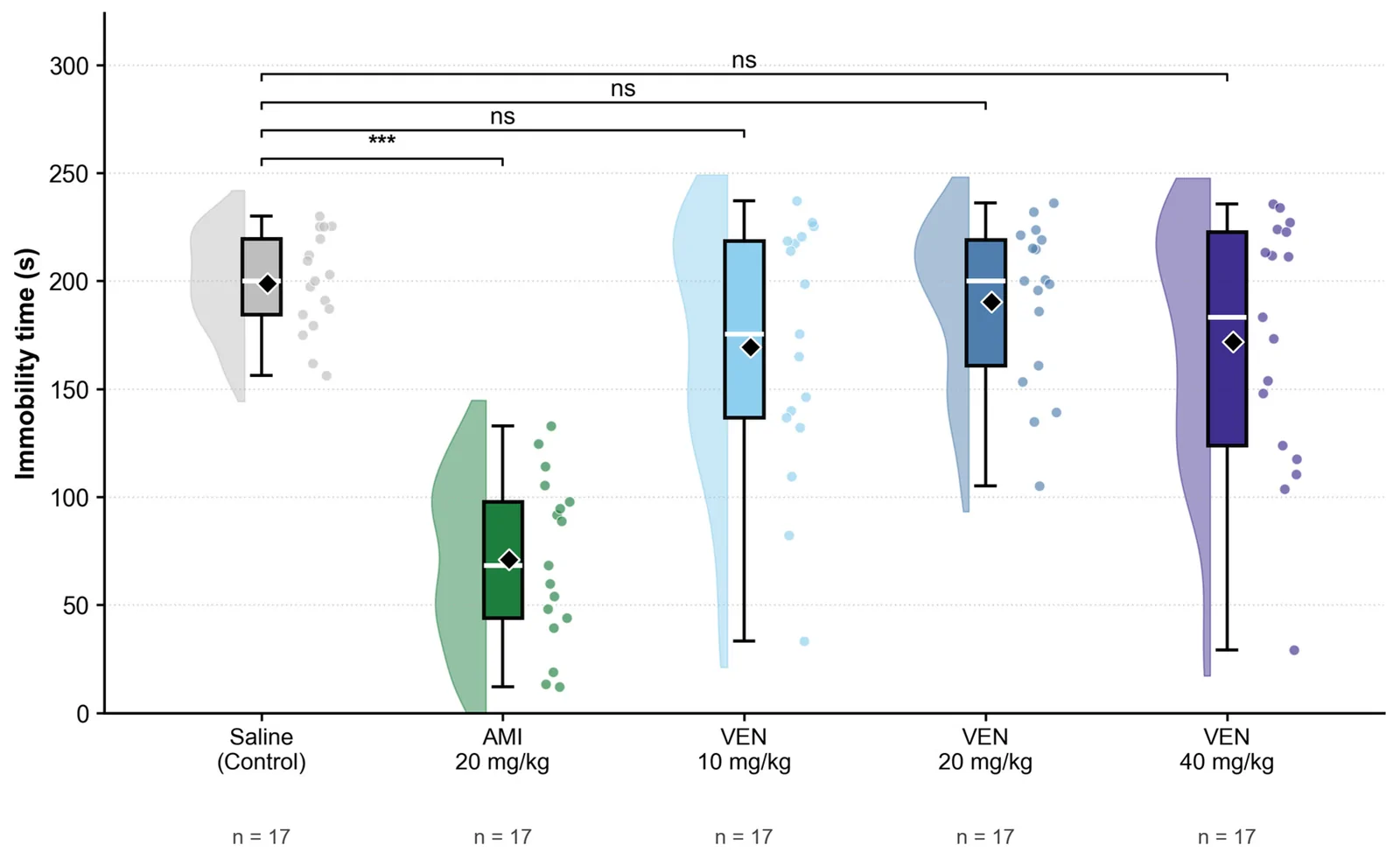
Effects of venlafaxine on immobility time in the forced swim test (FST). Immobility was scored during the final 4 min of a 6 min session. Raincloud plots show kernel-density distributions, boxplots (median and interquartile range), individual data points and group means (black diamonds). Brackets indicate Dunnett-adjusted comparisons versus saline-treated controls (*** *p* < 0.001; ns, not significant). *n* = 17 per group. AMI, amitriptyline; VEN, venlafaxine.

In Experiment 1 (FST, *n* = 17 per group; Figure 1), treatment significantly affected immobility time (one-way ANOVA *F*(4, 80) = 21.58, *p* < 0.001, η^2^ = 0.52, and ω^2^ = 0.49; Welch ANOVA *F*(4, 38.5) = 34.12, *p* < 0.001; Kruskal–Wallis *H*(4) = 34.72, *p* < 0.001, and ε^2^ = 0.38). This omnibus effect was driven entirely by the positive control. Amitriptyline (AMI) 20 mg/kg reduced immobility from 198.98 ± 22.79 s to 71.03 ± 38.68 s (−64.3%, bootstrap 95% CI −73.4 to −55.0; Dunnett *p* < 0.001; Hedges’ *g* = −3.94, 95% CI −5.11 to −2.76; Cliff’s δ = −1.00), confirming assay sensitivity. Descriptive statistics and assumption testing are summarised in Supplementary Table S1.

VEN produced no significant reduction at any dose. Mean immobility times were 169.34 ± 58.57 s for VEN 10 mg/kg (−14.9%; Dunnett *p* = 0.188; *g* = −0.65, 95% CI −1.34 to 0.04), 190.36 ± 38.27 s for VEN 20 mg/kg (−4.3%; *p* = 0.951; *g* = −0.27) and 171.89 ± 59.26 s for VEN 40 mg/kg (−13.6%; *p* = 0.254; *g* = −0.59). Tukey- and Dunn–Bonferroni-adjusted comparisons were concordantly non-significant (all *p* ≥ 0.33 and *p* = 1.00, respectively; Supplementary Table S2). There was no monotonic dose–response relationship across the 0–40 mg/kg range (Spearman ρ = −0.085, *p* = 0.49), and Bayes factors provided no evidence for an effect at any dose (*BF*_10_ = 1.36, 0.42 and 1.06 for 10, 20 and 40 mg/kg). Behavioural responses were variable, particularly at 10 and 40 mg/kg (Figure 1).

### 2.2. Venlafaxine Reduces Immobility in the Tail Suspension Test in a Dose-Dependent Manner

**Figure 2:**
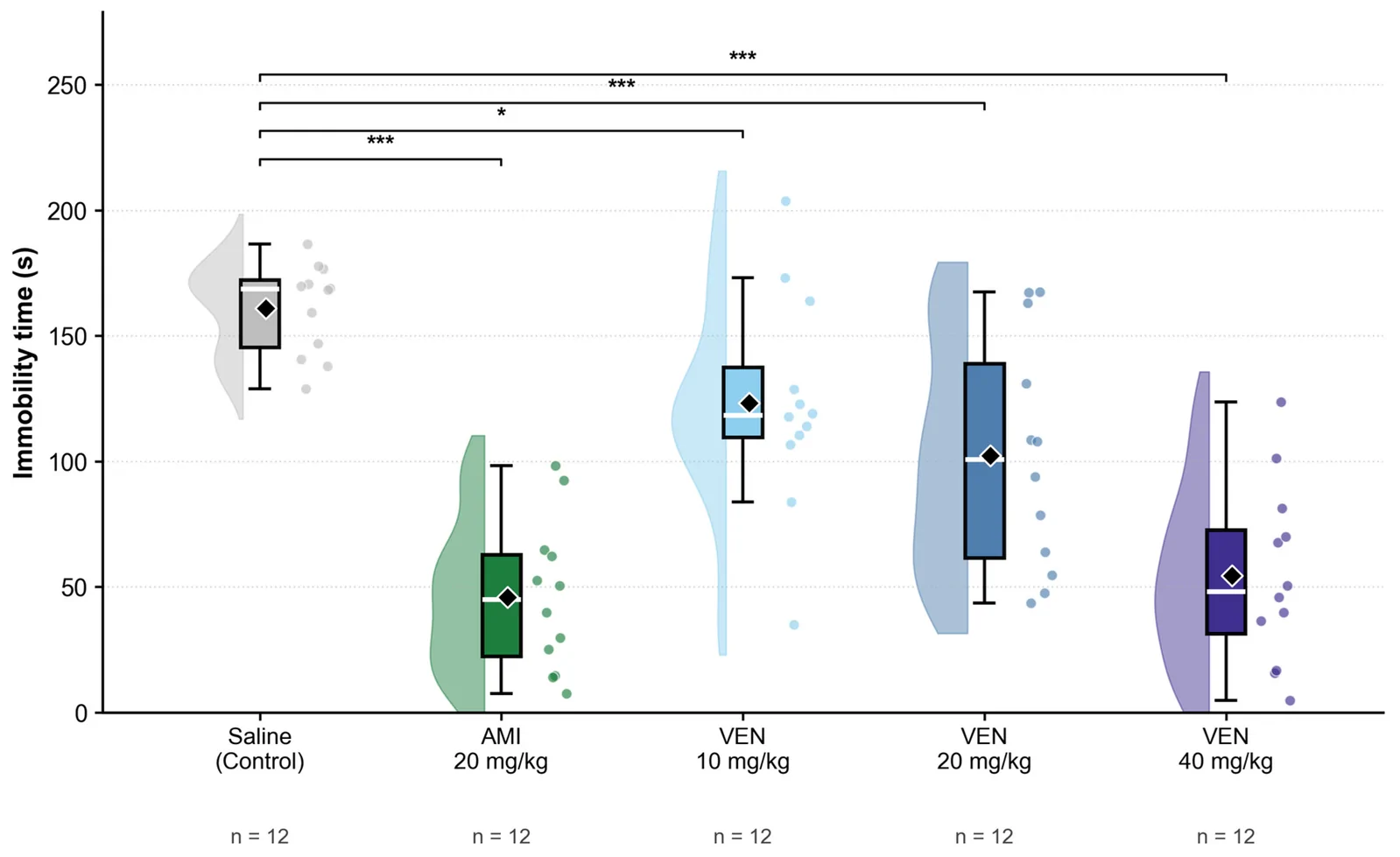
Effects of venlafaxine on immobility time in the tail suspension test (TST). Immobility was accumulated over the 6 min session. Raincloud plots show kernel-density distributions, boxplots (median and interquartile range), individual data points and group means (black diamonds). Brackets indicate Dunnett-adjusted comparisons versus saline-treated controls (* *p* < 0.05; *** *p* < 0.001). *n* = 12 per group. AMI, amitriptyline; VEN, venlafaxine.

In Experiment 2 (TST, *n* = 12 per group; Figure 2), treatment significantly affected immobility time (one-way ANOVA *F*(4, 55) = 21.14, *p* < 0.001, η^2^ = 0.61, and ω^2^ = 0.57; Welch ANOVA *F*(4, 26.4) = 40.94, *p* < 0.001; Kruskal–Wallis *H*(4) = 36.28, *p* < 0.001, and ε^2^ = 0.59). AMI 20 mg/kg reduced immobility from 161.03 ± 18.21 s to 45.93 ± 29.87 s (−71.5%, 95% CI −81.2 to −60.9; Dunnett *p* < 0.001; *g* = −4.49).

In contrast to the FST, VEN produced a graded, monotonic reduction across the dose range (Spearman ρ = −0.736, *p* < 0.001). VEN 10 mg/kg reduced immobility to 123.23 ± 43.25 s (−23.5%, 95% CI −38.8 to −7.9; *g* = −1.10, 95% CI −1.96 to −0.24; *BF*_10_ = 4.91). This effect was significant under Dunnett correction (*p* = 0.045) but not under the more conservative Tukey (*p* = 0.093) or Dunn–Bonferroni (*p* = 0.737) procedures and is therefore reported as borderline. VEN 20 mg/kg reduced immobility to 102.27 ± 46.62 s (−36.5%, 95% CI −52.3 to −20.0; Dunnett *p* < 0.001; Tukey *p* = 0.002; *g* = −1.60, 95% CI −2.53 to −0.67; *BF*_10_ = 52.3) and VEN 40 mg/kg to 54.42 ± 35.85 s (−66.2%, 95% CI −77.9 to −53.7; Dunnett *p* < 0.001; *g* = −3.62, 95% CI −4.96 to −2.28; *BF*_10_ = 1.3 × 10^6^). VEN 40 mg/kg did not differ from AMI (Tukey *p* = 0.978; Welch *p* = 0.535; *g* = +0.25, 95% CI −0.55 to 1.05; *BF*_10_ = 0.43), indicating an effect of similar magnitude at the doses tested; the absence of a significant difference does not establish equivalence between the two treatments.

The dissociation between the two assays was supported by a linear model on control-standardised immobility scores from Experiments 1 and 2 (87 animals; Section 4.6), which revealed a significant test × dose interaction (*F*(1,83) = 9.45, *p* = 0.003) in addition to the main effects of the test (*F*(1,83) = 30.10, *p* < 0.001) and dose (*F*(1,83) = 7.36, *p* = 0.008). VEN 20 mg/kg was therefore selected as the submaximal dose for the combination experiments, since it produced a reliable but non-maximal effect (−36.5%), leaving adequate dynamic range to detect either potentiation or attenuation.

Effect-size estimates for all experimental series are summarised in Supplementary Table S3.

### 2.3. Codeine Does Not Potentiate the Effect of Venlafaxine in the Tail Suspension Test

**Figure 3:**
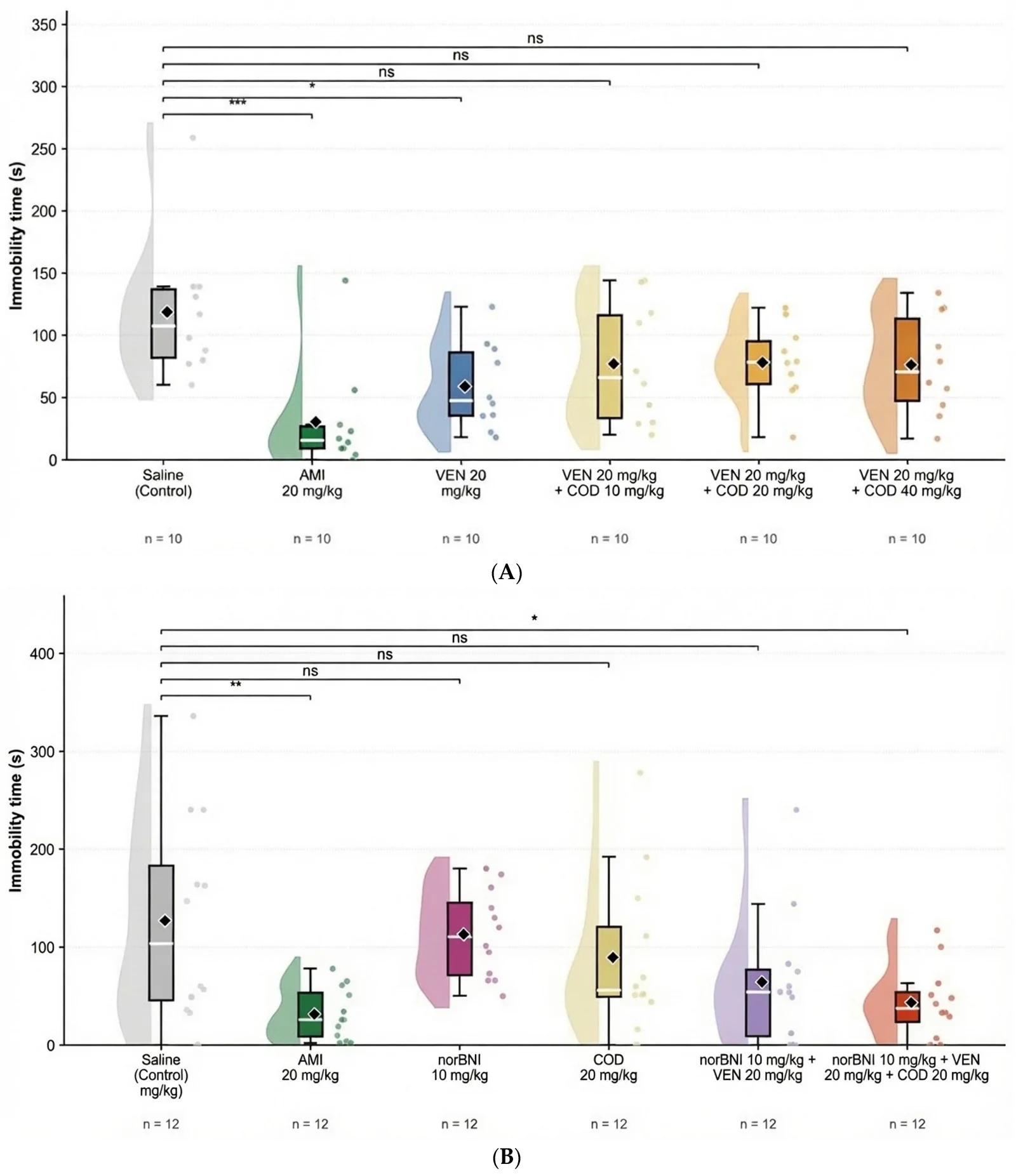
Effects of combined drug administration on immobility time in the tail suspension test (TST). (**A**) Effects of venlafaxine (VEN, 20 mg/kg) combined with codeine (COD, 10, 20, and 40 mg/kg). (**B**) Effects of combined nor-binaltorphimine, venlafaxine, and codeine administration. Immobility time was accumulated over the 6 min session. Raincloud plots show kernel-density distributions, boxplots (median and interquartile range), individual data points, and group means (black diamonds). Brackets indicate Dunnett-adjusted comparisons versus saline-treated controls ((**A**): * *p* < 0.05, *** *p* < 0.001; (**B**): * *p* < 0.05, ** *p* < 0.01; ns, not significant). Sample sizes were *n* = 10 per group for (**A**) and *n* = 12 per group for (**B**). AMI, amitriptyline; COD, codeine; norBNI, nor-binaltorphimine; VEN, venlafaxine.

In Experiment 3 (TST, *n* = 10 per group; Figure 3A), VEN 20 mg/kg was combined with COD in geometric progression (10, 20 and 40 mg/kg). Treatment significantly affected immobility time (one-way ANOVA *F*(5, 54) = 4.50, *p* = 0.002, η^2^ = 0.29, and ω^2^ = 0.23; Welch ANOVA *F*(5, 25.0) = 3.28, *p* = 0.021; Kruskal–Wallis *H*(5) = 18.16, *p* = 0.003). AMI 20 mg/kg reduced immobility from 118.80 ± 56.49 s to 30.40 ± 42.96 s (−74.4%; Dunnett *p* < 0.001; *g* = −1.69), and VEN 20 mg/kg alone reproduced its Experiment 2 effect (58.90 ± 34.87 s; −50.4%, 95% CI −69.0 to −24.4; Dunnett *p* = 0.013; *g* = −1.22).

Adding COD did not enhance this effect at any dose. Mean immobility values were 77.00 ± 48.03 s for VEN 20 + COD 10 (−35.2% vs. control; Dunnett *p* = 0.129), 78.20 ± 30.74 s for VEN 20 + COD 20 (−34.2%; *p* = 0.146) and 76.20 ± 40.11 s for VEN 20 + COD 40 (−35.9%; *p* = 0.118). None of the three combinations differed significantly from saline. Relative to VEN 20 mg/kg alone, the addition of COD was associated with a numerical increase in immobility of 30.7%, 32.8% and 29.4% at 10, 20 and 40 mg/kg, respectively; none reached significance (Welch *p* = 0.349, 0.206 and 0.317; *g* = +0.41, +0.56 and +0.44), and the Bayes factor for the most pronounced comparison indicated no evidence of a difference (VEN 20 + COD 20 vs. VEN 20, *BF*_10_ = 0.72). The attenuation showed no dependence on COD dose across the eightfold range examined (Spearman ρ = 0.152, *p* = 0.349), arguing against a dose-graded antagonism and indicating instead a flat, near-maximal effect already present at 10 mg/kg.

### 2.4. The Triple Combination of Venlafaxine, Codeine and Nor-Binaltorphimine Reduces Immobility in the Tail Suspension Test

Experiment 4 (TST, *n* = 12 per group; Figure 3B) compared the triple regimen norBNI 10 mg/kg + VEN 20 mg/kg + COD 20 mg/kg with norBNI alone, COD alone, the binary combination norBNI + VEN and AMI. Treatment significantly affected immobility time (one-way ANOVA *F*(5, 66) = 3.99, *p* = 0.003, η^2^ = 0.23, and ω^2^ = 0.17; Welch ANOVA *F*(5, 29.9) = 7.13, *p* < 0.001; Kruskal–Wallis *H*(5) = 18.70, *p* = 0.002).

Neither monotherapy was effective: norBNI 10 mg/kg produced 113.00 ± 44.76 s (−11.1%; Dunnett *p* = 0.980; *g* = −0.17; *BF*_10_ = 0.40), and COD 20 mg/kg produced 89.50 ± 80.51 s (−29.6%; *p* = 0.501; *g* = −0.39; *BF*_10_ = 0.53), both against a control value of 127.08 ± 104.88 s. The binary combination norBNI + VEN 20 mg/kg reduced immobility to 64.25 ± 69.60 s (−49.4%, 95% CI −77.5 to 5.4; *g* = −0.68), which did not reach significance (Dunnett *p* = 0.091; *BF*_10_ = 1.07).

By contrast, the triple combination reduced immobility to 43.58 ± 36.43 s (−65.7%, 95% CI −82.4 to −33.2; Dunnett *p* = 0.013; *g* = −1.03, 95% CI −1.88 to −0.17; Cliff’s δ = −0.53; *BF*_10_ = 3.63). The magnitude was comparable to AMI 20 mg/kg (31.50 ± 26.49 s; −75.2%; Dunnett *p* = 0.004; *g* = −1.21), and the two did not differ from one another (Welch *p* = 0.364; Mann–Whitney *p* = 0.563; *BF*_10_ = 0.51). The triple combination was clearly superior to norBNI monotherapy (Welch *p* < 0.001; Mann–Whitney *p* = 0.001, Holm-adjusted *p* = 0.004; *g* = −1.64, 95% CI −2.58 to −0.71; *BF*_10_ = 63.9).

Three findings temper this interpretation. First, the triple combination did not differ significantly from the binary norBNI + VEN regimen (Welch *p* = 0.375; Mann–Whitney *p* = 0.468; *g* = −0.36, 95% CI −1.17 to 0.45; *BF*_10_ = 0.51), so the incremental contribution of COD within the triple regimen cannot be established from these data. Second, under the most conservative non-parametric correction, the effect of the triple combination versus saline did not survive (Dunn–Bonferroni *p* = 0.287), reflecting the low power of rank-based tests with fully corrected multiplicity at *n* = 12 per group. Third, the saline control of Experiment 4 was unusually dispersed (127.08 ± 104.88 s) and differed appreciably from the controls of the other experiments, so any comparison with treatments tested in a different cohort, including VEN 20 mg/kg alone and VEN 20 mg/kg + COD 20 mg/kg from Experiment 3, is indirect and is made with caution. The non-significant results for norBNI alone, COD alone and norBNI + VEN were obtained with group sizes that were underpowered for intermediate effects and do not demonstrate the absence of an effect. The parametric Dunnett result, the effect-size estimate and the sensitivity analyses reported in Section 2.5 nonetheless converge on a reproducible effect of the triple combination relative to its own control.

### 2.5. Sensitivity Analyses Confirm the Robustness of the Triple-Combination Effect

The saline group in Experiment 4 showed unusually high dispersion (SD = 104.88 s; range 0–336 s), which warranted formal examination. No observation qualified as an outlier by the Tukey criterion (1.5 × IQR limits: −160.1 to 388.9 s), and in accordance with the pre-specified protocol, no animal or data point was excluded. Three pre-planned sensitivity analyses were therefore performed.

Symmetric 10% winsorisation strengthened rather than weakened the findings: the omnibus effect increased (*F*(5, 66) = 5.93, *p* < 0.001), and the triple combination remained significant with a smaller *p*-value (Dunnett *p* = 0.003), while the binary norBNI + VEN combination became significant (*p* = 0.018). Welch’s heteroscedasticity-robust ANOVA likewise yielded a stronger omnibus result than the standard ANOVA (*p* < 0.001 versus *p* = 0.003). Conversely, logarithmic transformation attenuated all contrasts (triple combination *p* = 0.222), but this transformation is inappropriate here because five groups contained zero values, which log(x + 1) compresses disproportionately; it is reported for completeness only.

Observed statistical power for the omnibus tests was high in all four experiments (1 − β = 1.00, 1.00, 0.97 and 0.95 for Experiments 1–4, respectively). For the key pairwise contrast, the triple combination versus saline, observed power was 0.67 at *n* = 12 per group; approximately 16 animals per group would be required for 80% power at the observed effect size. The binary norBNI + VEN contrast was clearly underpowered (observed power 0.36; approximately 35 animals per group required), which explains why a −49.4% reduction did not reach significance and reinforces that its non-significance should not be read as evidence of absence. The minimum detectable standardised effect at *n* = 12 per group and 80% power was |d| ≈ 1.20.

### 2.6. Cross-Experimental Synthesis

Numerical outcomes for all four experimental series are presented in Table 1, and the corresponding pharmacodynamic interpretation is summarised in Table 2.

All values were computed directly from the raw data. Percentage change and its 95% confidence interval (CI) refer to the group mean relative to the saline control of the same experiment; CIs were obtained by bootstrap resampling (20,000 resamples). Hedges’ g is the bias-corrected standardised mean difference versus the control, with negative values indicating reduced immobility. Cliff’s δ magnitude thresholds are as follows: negligible < 0.147, small < 0.33, medium < 0.474 and large ≥ 0.474. *p* values are from Dunnett’s test versus saline; complete Tukey and Dunn–Bonferroni matrices are given in Supplementary Table S2. AMI, amitriptyline; COD, codeine; FST, forced swim test; IQR, interquartile range; norBNI, nor-binaltorphimine; SD, standard deviation; TST, tail suspension test; VEN, venlafaxine.

**Figure 4:**
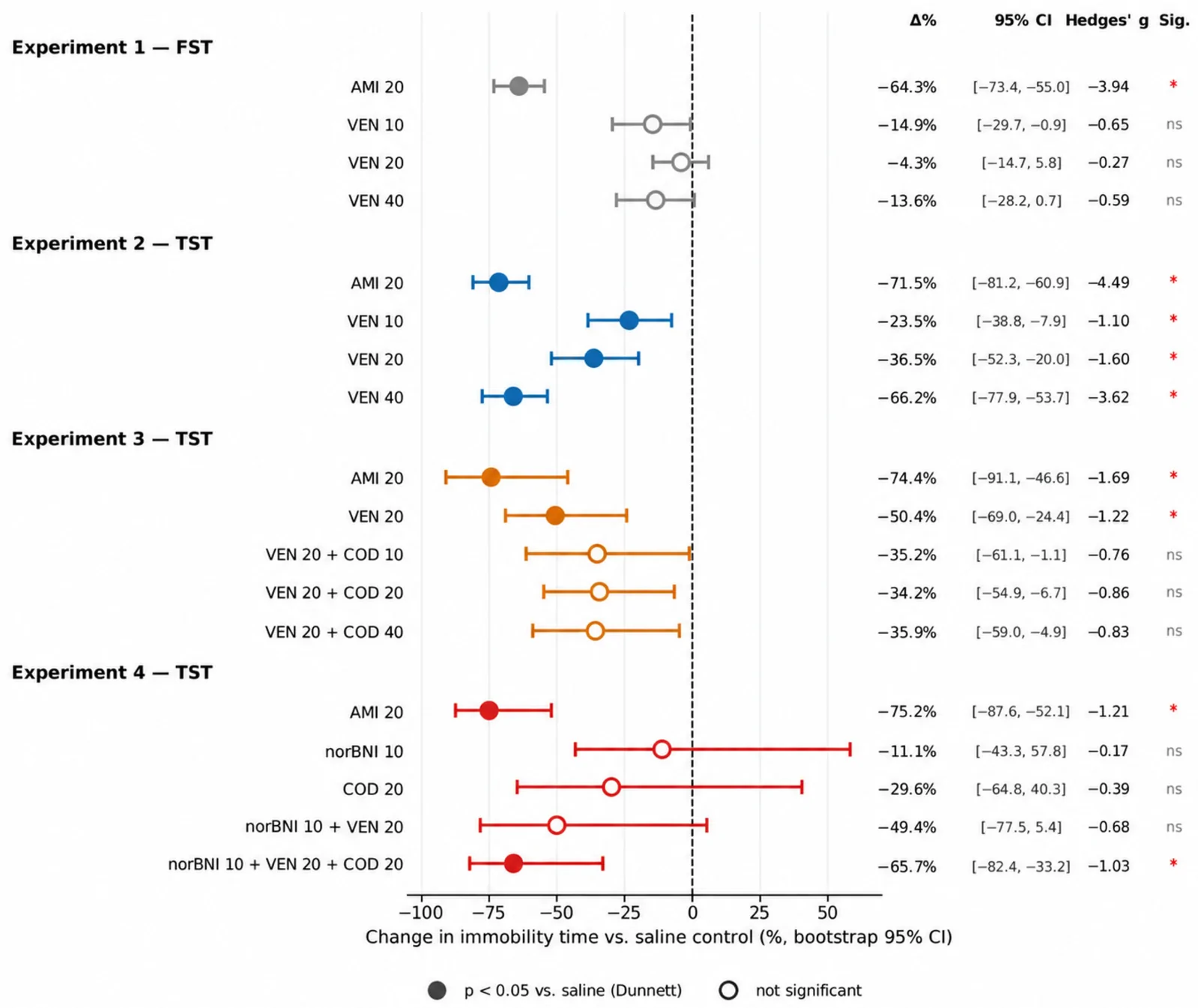
Cross-experimental synthesis of antidepressant-like effects. Forest plot of the percentage change in immobility time relative to the saline control for every treatment group across the four experiments, with bootstrap 95% confidence intervals (20,000 resamples). The asterisk (*) indicates comparisons significant at *p* < 0.05 versus saline (Dunnett); open circles denote non-significant comparisons. Right-hand columns give the point estimate, its 95% confidence interval and Hedges’ g. Arrowheads indicate confidence intervals extending beyond the plotted range. AMI, amitriptyline; COD, codeine; FST, forced swim test; norBNI, nor-binaltorphimine; TST, tail suspension test; VEN, venlafaxine; ns, not significant.

Sensitivity analyses for Experiment 4 are detailed in Table 3, and Figure 4 displays the percentage change in immobility with bootstrap 95% confidence intervals for every treatment group across the four experiments.

The saline group of Experiment 4 showed high dispersion (SD = 104.88 s), so three pre-specified sensitivity analyses were applied. No observation met the Tukey 1.5 × IQR outlier criterion, and no data were excluded. Winsorisation and Welch correction strengthened the primary result; the logarithmic transformation is reported for completeness but is inappropriate here because five groups contained zero values. AMI, amitriptyline; ANOVA, analysis of variance; COD, codeine; IQR, interquartile range; norBNI, nor-binaltorphimine; SD, standard deviation; VEN, venlafaxine.

The overall pattern is consistent. In the TST, monoaminergic enhancement alone produced a dose-dependent effect; µ-opioid agonism added to a submaximal SNRI dose produced no benefit; and the combination of κ blockade with SNRI and µ-opioid agonism produced an effect similar in magnitude to that of amitriptyline, which none of the single treatments tested in the same cohort reached. Because the additivity benchmarks computed from the component effects (−64.4% under a Bliss independence model; −79.0% under simple additivity) bracket the observed −65.7%, the data are consistent with additive-to-cooperative interaction and do not demonstrate supra-additivity. Because the triple combination did not differ significantly from norBNI + VEN, these data do not evidentiate a specific contribution of codeine to the combined effect but also do not exclude it.

### 2.7. Molecular Docking Is Consistent with the Target Assignment Underlying the Combination

The protocol was validated before the study compounds were examined by returning each co-crystallised ligand to its own receptor under identical settings (Table 4). All eight structures passed: the top-ranked mode reproduced the experimental geometry with a median heavy-atom deviation of 0.73 Å and a worst case of 1.39 Å, inside the 2.0 Å criterion defined a priori. Two independent checks used ligands rebuilt from their chemical structures rather than lifted from the coordinates: amitriptyline was placed in the noradrenaline transporter within 0.75 Å of its experimental position, marginally closer than the 0.88 Å obtained when the same ligand was returned from the deposited coordinates, and morphine was placed in the μ-opioid receptor with its protonated nitrogen 3.10 Å from Asp149, in the top-ranked mode.

RMSD, symmetry-corrected heavy-atom root-mean-square deviation of the top-ranked mode from the crystallographic pose, was computed in the experimental frame without superposition and minimised over all graph automorphisms of the ligand. The anchor distance is the shortest separation, in the deposited structure, between a ligand nitrogen and a carboxylate oxygen of the conserved aspartate of transmembrane helix 3. All eight structures are human. We used AutoDock Vina 1.2.5, exhaustiveness 32.

Poses were then examined for the interaction that anchors every cationic ligand of these two protein families, namely the ion pair between the protonated ligand nitrogen and the conserved aspartate of transmembrane helix 3, which is Asp98 in SERT, Asp75 in NET, Asp79 in DAT and Asp3.32 in each opioid receptor. The criterion was taken from the structures themselves. In six of the eight complexes, this distance lies between 2.62 and 3.40 Å. Two structures fall outside it: the noradrenaline transporter, where the amitriptyline amine sits 4.46 Å from Asp75, and the inactive μ-opioid receptor, where the deposited naloxone sits 5.71 Å from Asp149. A cut-off of 4.0 Å was adopted, and both the top-ranked mode and the best-scoring anchor-consistent mode are reported (Table 5), with the interaction inventory of each ligand at its primary target given in Table 6. Because the Vina score scales with molecular size, ligand efficiency is reported alongside it. In the inactive μ-opioid receptor, the criterion is not informative, since the experimental ligand itself does not satisfy it, and no anchor-consistent mode was found there for any compound; only the top-ranked affinities are given for that structure.

VEN occupied the central S1 site of all three monoamine transporters. In SERT, its protonated dimethylamino group formed the ion pair with Asp98 at 3.86 Å in the top-ranked mode, the methoxyphenyl ring stacked against Tyr176, the ammonium group made a cation–π contact with Tyr95, and the cyclohexanol hydroxyl approached the backbone carbonyl of Phe335 at 3.18 Å, a set of contacts spanning subsites A, B and C of the orthosteric pocket. Anchor-consistent affinities were −7.95 kcal mol^−1^ at SERT, −7.70 at NET and −8.55 at DAT. Across the opioid receptors VEN scored between −5.62 and −6.97 kcal mol^−1^, below its value at any transporter and below that of each opioid ligand at its own receptor.

COD was accommodated in the orthosteric message–address pocket of the active-state μ-opioid receptor, the morphinan nitrogen lying 3.59 Å from Asp149, the aromatic system flanked by Trp295 and Tyr328 and the phenolic ring stacked on His299. Its anchor-consistent affinity was −7.68 kcal mol^−1^ at the μ receptor, against −6.70 and −5.89 at the two κ structures and −7.15 at the δ receptor, so the μ subtype was its preferred opioid target, which is the ordering its pharmacology requires. Morphine, docked as an internal reference, ranked the μ receptor first across the entire panel (−7.99 kcal mol^−1^) and reached −7.06 at the δ and −6.69 at the κ receptor.

norBNI behaved as its size and bivalent architecture predict. It was excluded from all three monoamine transporters, since no mode satisfying the anchoring criterion appeared among the twenty retained at any of them, and the unfiltered scores at SERT and NET, −3.49 and −4.16 kcal mol^−1^, are those of a ligand that cannot be housed in the S1 site. At the κ receptor, the top-ranked mode of both structures reproduced the bivalent message–address binding mode in full: one protonated morphinan nitrogen engaged Asp138 of transmembrane helix 3 (3.26 Å in the G_i_-coupled complex and 3.00 Å in the inactive receptor), while the second, carried on the pyrrole-linked unit, formed an independent ion pair with Glu297 of the address region, at 3.55 and 3.44 Å, respectively. That second contact has no counterpart at the other two subtypes because position 6.58 is Trp284 in the δ receptor and Lys305 in the μ receptor; in both, the docked pose left the second ammonium group without a partner. The scores were −10.20 kcal mol^−1^ at the G_i_-coupled κ receptor, −9.89 at the inactive κ receptor, −9.98 at the δ receptor and −10.14 at the active μ receptor.

Values in kcal mol^−1^ are the affinity of the best-scoring mode that satisfies the anchoring ion pair with the conserved aspartate of transmembrane helix 3 (≤4.0 Å). n.a. means that no anchor-consistent mode was found among the twenty retained; the affinity of the top-ranked mode is then given in square brackets. Top-ranked affinities for every cell, as the mean ± SD of three independent runs with fixed random seeds (SD ≤ 0.54 throughout), are given in Supplementary Table S4. In the asterisked structure (9PXU), the deposited naloxone lies 5.71 Å from Asp149, so the anchor criterion is not informative there, and only top-ranked values are available. The Vina score is an empirical ranking function and is not a thermodynamic binding free energy; it scales with molecular size and is therefore not comparable between ligands of different mass.

Three discordances are reported without adjustment. First, within the transporter family, the score did not follow the measured affinity order, since VEN was scored most favourably at DAT, the transporter for which its affinity is by far the weakest, and least favourably at SERT, its principal target. Second, the two morphinans are over-scored at the noradrenaline transporter: before the anchor filter COD reached −9.77 kcal mol^−1^ there, which the filter reduced to −7.86, a value that still edges out its μ-opioid score of −7.68 even though codeine has no meaningful affinity for this transporter. Third, although the κ-selective binding mode of norBNI was reproduced exactly, the score itself did not separate the κ receptor from the δ receptor or from the active μ receptor, the three lying within 0.3 kcal mol^−1^ of one another. The same size dependence is visible in ligand efficiency, which is 0.21 kcal mol^−1^ per heavy atom for norBNI against 0.40 for VEN, 0.42 for AMI and 0.35 for COD. All three observations reflect the known behaviour of empirical scoring functions, which reproduce binding geometry considerably better than they rank affinity between different pockets. The structural conclusion on which the present study rests is unaffected by them: each of the three agents engages a different site, and no pocket is engaged by more than one of them through the canonical anchoring interaction.

**Figure 5:**
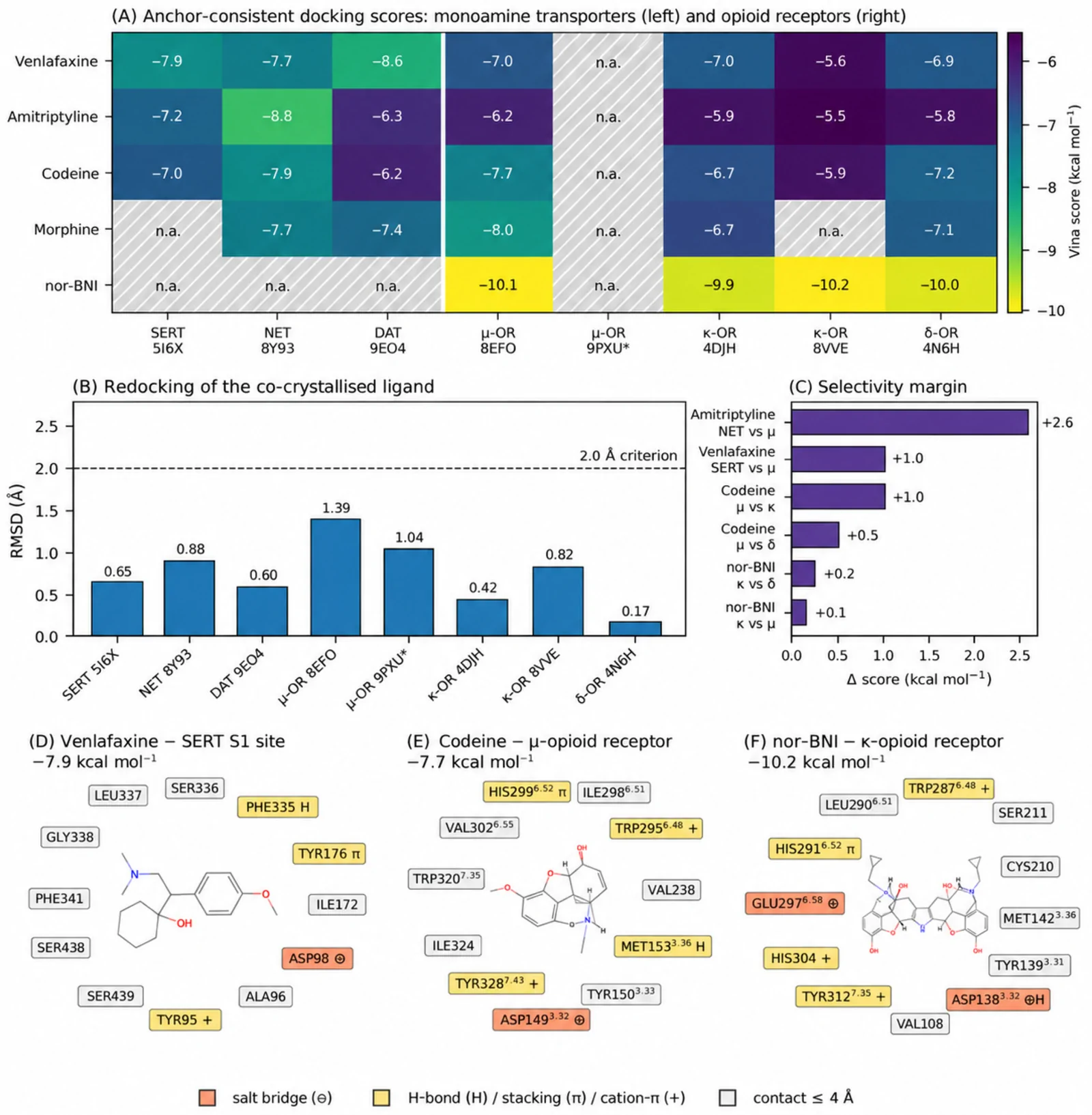
Molecular docking of venlafaxine, codeine and nor-binaltorphimine at their candidate targets. (**A**) Anchor-consistent docking scores for the five ligands across the eight experimental structures, all of them human; the white rule separates the monoamine transporters from the opioid receptors, and hatched cells mark pairs for which no anchor-consistent mode was retained. (**B**) Redocking of each co-crystallised ligand into its own receptor; bars show the symmetry-corrected heavy-atom deviation of the top-ranked mode and the dashed line the 2.0 Å criterion. (**C**) Selectivity margins, expressed as the difference in anchor-consistent score between the principal target and the indicated comparator; positive values favour the principal target. (**D**–**F**) Contact maps of the three complexes central to the combination, showing residues within 4.0 Å of the ligand in the best-scoring anchor-consistent pose, with opioid-receptor residues additionally labelled in Ballesteros–Weinstein notation. The asterisked (*) structure (9PXU) is the one in which the anchor criterion is not informative.

LE, ligand efficiency, is defined as the negative of the docking score divided by the number of heavy atoms. Salt bridges are cationic ligand nitrogen to carboxylate oxygen within 4.0 Å; hydrogen bonds require a donor–acceptor distance ≤ 3.5 Å and a donor–hydrogen–acceptor angle ≥ 120°; aromatic contacts comprise ring-centroid separations ≤ 5.5 Å and cation–π contacts ≤ 6.0 Å. The last column gives the total number of residues with a heavy atom within 4.0 Å of the ligand. The molecular docking results, including anchor-consistent docking scores, redocking validation, selectivity margins, and ligand–receptor interaction maps, are summarized in Figure 5. Affinities for all forty ligand–structure pairs are given in Supplementary Table S4.

## 3. Discussion

This study asked whether simultaneous engagement of complementary monoaminergic and opioid mechanisms, namely SNRI-mediated monoaminergic enhancement, sub-effective µ-opioid agonism and sustained κ-opioid antagonism, generates antidepressant-like activity exceeding that of the individual components. Four findings emerged. First, VEN failed to reduce FST immobility at 10–40 mg/kg despite a robust AMI response, with no dose–response trend whatsoever. Second, VEN produced a clear, monotonic dose-dependent reduction in TST immobility over the same dose range, reaching an effect of similar magnitude to that of AMI at 40 mg/kg. Third, adding COD in geometric progression did not potentiate VEN 20 mg/kg and was associated with a consistent but non-significant attenuation that did not vary with COD dose. Fourth, the triple combination of norBNI, VEN and COD produced a significant reduction in immobility, of similar magnitude to that of AMI, whereas none of the single treatments tested in the same cohort reached significance.

### 3.1. Dissociation Between the Forced Swim and Tail Suspension Tests

The divergence between assays deserves careful consideration because it was not a matter of degree but of kind: the FST showed no dose–response relationship at all (ρ = −0.085, *p* = 0.49), whereas the TST showed a strong monotonic one (ρ = −0.736, *p* < 0.001), and the test × dose interaction was statistically significant. Both paradigms rely on inescapable stress, but they differ substantially in motor demand, thermoregulatory load, behavioural strategy and pharmacological sensitivity. The murine TST is generally more responsive to serotonergic and dual-action antidepressants, whereas the FST shows greater strain-dependence and procedural variability that can obscure effects of intermediate magnitude [59,66,67]. Our data are consistent with this literature and extend it by quantifying the dissociation within a single strain, laboratory and dose range.

A second contributing factor is the interaction between monoaminergic activation and immobility-based scoring. Compounds with substantial noradrenergic activity may alter vigilance and locomotor output independently of any effect on affective state, and reductions in immobility therefore do not necessarily reflect changes in the thymic state of the animal alone [13,14]. This is particularly relevant for VEN, whose behavioural profile is strongly dose- and context-dependent [7,11]. It is worth emphasising, however, that a locomotor confound would be expected to produce a spurious reduction in immobility rather than the absence of an effect we observed in the FST; the FST null result is therefore unlikely to be explained by motor activation and more plausibly reflects genuinely lower assay sensitivity to SNRIs in NMRI mice. The absence of an independent locomotor control remains a limitation (Section 3.6). Overall, these findings reinforce the value of employing complementary behavioural assays when characterising compounds that combine serotonergic and noradrenergic mechanisms [65,67]. These observations suggest a testable explanation for the dissociation rather than a general ranking of the two assays. The FST imposes a thermoregulatory and energetic load that the TST does not, and immobility is scored over a shorter window after a stabilisation period, so a serotonergic-preferring drug may redistribute active behaviours without reducing total immobility. This hypothesis yields three directly testable predictions: (1) that venlafaxine increases active behaviours in the FST without changing total immobility when swimming and climbing are scored separately, as opioid-type and monoaminergic response patterns are separated in the TST [55]; (2) that reducing the thermoregulatory load by raising water temperature increases the sensitivity of the FST to venlafaxine; and (3) that the dissociation persists when a concurrent locomotor control is included. Until these experiments are performed, we restrict the conclusion to the statement that, under the conditions of this study, the TST was more sensitive to venlafaxine than the FST.

### 3.2. Codeine Does Not Potentiate Venlafaxine: A Pharmacologically Coherent Negative Result

COD combined with SSRIs enhances antidepressant-like activity in mice, whereas the same combination with the noradrenaline reuptake inhibitor desipramine or with the SNRI duloxetine produces no potentiation [54]. Our result aligns precisely with that pharmacological selectivity: VEN at 20 mg/kg, a dose at which noradrenergic recruitment is already substantial [7,8], behaved like the SNRI arm rather than the SSRI arm of that study. The convergence is notable because it was obtained using a different laboratory, strain and dose regimen, and it strengthens the inference that the cooperative COD–monoamine interaction is specifically serotonergic. Comparable serotonergic selectivity has been described for 5-HT_3_ receptor antagonists, which augment fluoxetine and venlafaxine but not desipramine [68,69].

Several non-exclusive mechanisms may contribute. Because VEN 20 mg/kg already produced a substantial effect (−50.4% in Experiment 3), the dynamic range available for further improvement was reduced, although not exhausted: AMI achieved −74.4% in the same experiment, so a ceiling artefact alone is an insufficient explanation. More informative is the flatness of the response across an eightfold COD dose range (ρ = 0.152, *p* = 0.349): a competitive or dose-graded antagonism would be expected to deepen with dose, whereas a saturable counter-regulatory process would not.

A counter-regulatory process is one pharmacologically plausible explanation. Sustained µ-opioid receptor activation upregulates dynorphin [70,71], and µ and κ receptors exert functionally opposing actions within shared limbic and striatal circuits [72], so the net behavioural outcome of VEN + COD may reflect the balance between positive affective µ-opioid signalling and dynorphin-mediated negative feedback [35,36]. We present this only as a hypothesis for two reasons: the evidence for µ-agonist-driven dynorphin upregulation comes mainly from spinal [70] and dorsal striatal [71] preparations rather than from the mesolimbic circuits relevant to affective behaviour, and the present study measured no neurochemical variable that could test it. Its value here is that it makes a directional prediction, namely that removing κ-mediated feedback should unmask the µ-opioid contribution, and Experiment 4 was designed to test that prediction.

### 3.3. The Triple Combination: Magnitude of the Effect and Limits of Interpretation

The behavioural effect in Experiment 4 emerged only when all three mechanisms were engaged concurrently. κ blockade alone produced no significant change (−11.1%), µ agonism alone produced no significant change (−29.6%), and their pairing with VEN produced a graded progression: norBNI + VEN −49.4% (*p* = 0.091) and norBNI + VEN + COD −65.7% (*p* = 0.013). This ordering is consistent with the hypothesis that removing dynorphin-mediated negative feedback creates a permissive neurobiological context in which monoaminergic and µ-opioid facilitation become behaviourally detectable [33,37,52]. It is also consistent with the observation that κ antagonism prolongs and stabilises the antidepressant-like action of other agents rather than producing an effect of its own [44].

We deliberately refrain from describing this pattern as synergy. Three considerations impose caution. First, the triple combination did not differ statistically from the binary norBNI + VEN regimen (*p* = 0.375; *BF*_10_ = 0.51), so the specific contribution of COD is not resolved; the ordering of the point estimates is suggestive but not evidential. Second, the observed −65.7% falls between the additivity benchmarks computed from the component effects (−64.4% under Bliss independence, −79.0% under simple additivity), which is the signature of an additive rather than supra-additive interaction. Third, formal demonstration of synergy requires isobolographic or response-surface analysis across multiple dose combinations of each component [73,74,75], which the present fixed-dose design cannot provide. What the data establish is narrower: a statistically significant antidepressant-like effect that was present for the triple combination and absent for each single treatment tested in the same cohort. Because the triple combination did not differ from norBNI + VEN, this pattern is equally compatible with an effect carried mainly by κ blockade together with the SNRI, and it does not show that all three components are required. We therefore use cooperativity only in the operational sense defined in the Introduction, and we claim neither potentiation nor synergy.

### 3.4. Structural Basis of the Multi-Target Design

The behavioural design rests on an assumption that behaviour itself cannot test, namely that each of the three agents engages the target set attributed to it and does not engage the others. The docking analysis addressed that assumption directly, and its verdict is affirmative with two qualifications that are best stated before the positive findings.

The protocol reproduced the geometry it was asked to reproduce. All eight co-crystallised ligands were returned to within 1.39 Å of their experimental positions, with a median deviation of 0.73 Å. Two more demanding tests gave the same answer: amitriptyline and morphine, rebuilt from their chemical structures rather than lifted from the coordinates, were placed in the noradrenaline transporter and in the μ-opioid receptor, respectively, in poses that reproduce the experimental binding mode, morphine with its protonated nitrogen 3.10 Å from Asp149 in the top-ranked mode [76]. The poses on which the following arguments rest are therefore not artefacts of the search.

Within that framework, the docking results are consistent with the intended target assignment. VEN occupied the central S1 site of all three monoamine transporters, and in SERT, its protonated dimethylamino group formed the ion pair with Asp98 that anchors every substrate and inhibitor of this transporter [77]. At the opioid receptors, VEN scored below its value at any transporter and below that of each opioid ligand at its own receptor, which is the expected pattern for a compound reported to be without measurable affinity at opioid binding sites [10]. COD, conversely, was accommodated in the orthosteric message–address pocket of the μ receptor, with the morphinan nitrogen engaging Asp149 and the aromatic system flanked by Trp295 and Tyr328, and it was less well accommodated at the κ and δ receptors. That ordering is the structural counterpart of the pharmacological observation that 3-O-methylation of morphine lowers opioid receptor affinity without altering the μ-over-δ and μ-over-κ ratios [22]. norBNI reproduced the behaviour expected of a bivalent morphinan: one pharmacophore in the message pocket at Asp138, the second forming an independent ion pair with Glu297 of the address region, the residue at position 6.58 that distinguishes the κ subtype and that is replaced by tryptophan in the δ receptor and by lysine in the μ receptor [78,79]. The essential point for the present study is negative and simple. Each agent engages a different site through the canonical anchoring interaction, no site is engaged by more than one of them, and nothing in the structural data indicates that the three would compete for a shared pocket at the doses used. The interaction observed in Experiment 4 is therefore more likely to arise downstream of target engagement, in the circuitry that these separate targets share, although docking alone cannot establish where, or whether, such an interaction takes place.

The two qualifications concern what an empirical scoring function can rank. First, the score did not reproduce the established affinity order within the transporter family, since VEN scored most favourably at the dopamine transporter, for which its measured affinity is by far the weakest [80]. Docking scores are dominated by shape complementarity and buried surface and are known to rank affinity poorly across targets even when they reproduce poses well [81,82]; we report the discordance rather than accommodate it and draw the corresponding conclusion that the calculation supports the identity of the sites occupied, not the relative affinity for them. Second, docking is a poor instrument for demonstrating that a compound does not bind: any protonated amine placed in an aminergic pocket will find the conserved aspartate, and VEN duly formed that contact at the μ and κ receptors, although it has no measurable affinity for either. What distinguishes those complexes is not the ion pair but the poverty of everything around it, so the reading must be comparative rather than absolute.

Taken together, the structural analysis is consistent with an explicit model of the combination: monoaminergic facilitation at the transporter S1 sites, a weak μ-opioid input at the morphinan pocket and removal of κ-mediated restraint at a third, non-overlapping site, with convergence occurring in mesolimbic and prefrontal circuitry rather than at any single protein.

### 3.5. Translational Perspective

These findings intersect with a substantial collection of clinical literature on opioid modulation in depression. Buprenorphine/samidorphan demonstrated adjunctive efficacy in inadequate responders [45], though subsequent phase 3 results were mixed [46], and selective κ antagonism improved reward responsiveness in the FAST-MAS proof-of-mechanism trial [47], while monotherapy and augmentation studies have yielded modest or inconclusive outcomes [48,49,50]. Our results suggest a possible reconciliation: κ blockade may be more useful as an enabling component within a multi-target regimen than as a standalone intervention, since in our hands it produced no significant effect when given alone yet was associated with the largest effects when combined.

Any translation must confront the fact that norBNI is not a viable clinical agent. Its extraordinarily persistent, JNK1-dependent pharmacodynamic action [41,42] complicates safety assessment and trial design [51,52,53]. A translationally realistic version of the present regimen would therefore pair a κ antagonist with human-compatible pharmacokinetics, such as aticaprant [47,50], with an SNRI and a µ-opioid modulator of low abuse liability. We note that the “low abuse potential” of COD is defensible only relative to morphine and is itself metabolite-dependent [22,83]; codeine misuse is well documented, and any clinical extension of this strategy would require a µ-opioid component with a more favourable liability profile. Sex hormones and oestrogen-receptor signalling also influence stress responsivity and monoaminergic neurotransmission, making sex-stratified evaluation essential before translation.

Two consequences for future clinical direction follow from these data, and both are modest. First, the pairing that carries most of the observed effect is κ-opioid blockade combined with an SNRI rather than the addition of a µ-opioid agonist, so the informative next preclinical step is a complete factorial design in a single cohort and not a further dose escalation of codeine. Second, because the effect was obtained in acute assays in naive animals, the clinical question these data inform is narrow, namely whether κ antagonism added to an SNRI improves the stress-coping and reward-related domains that such assays engage. A regimen of translational interest would therefore pair an SNRI with a κ antagonist suitable for human use, such as aticaprant [47,48], and would need to be evaluated in chronic stress models with anhedonia and effort-based outcomes, in both sexes and in more than one strain, before any inference about treatment-resistant depression is drawn.

### 3.6. Strengths and Limitations

The principal strengths of this study are its systematic four-step design, the inclusion of a positive control in every experiment, geometric dose progression, direct comparison of monotherapies with binary and triple combinations and a convergent analytical strategy combining parametric, non-parametric and Bayesian inference with multiple effect-size metrics and pre-specified sensitivity analyses. Blinded offline scoring and computer-generated randomisation reduce the risk of detection and selection bias.

The limitations are equally important. The study used a single strain and sex (male NMRI mice) and relied on acute immobility-based paradigms; generalisability to female animals, to chronic-stress models such as chronic mild stress, social defeat or learned helplessness and to behavioural domains such as anhedonia or motivational impairment remains to be established. The combination experiments were conducted exclusively in the TST, so the FST/TST dissociation was characterised only for VEN monotherapy. The FST and TST assess responses to acute inescapable stress and are simplified approximations of depressive-like states rather than models of human depression [63,64]; extrapolation to clinical efficacy is therefore indirect. The design is not a model of treatment resistance: animals were naive, unstressed and had not failed a previous antidepressant, so the treatment-resistant phenotype is the clinical target of the strategy rather than a feature of the assay.

No independent locomotor assessment was performed, and this is an important limitation of the study. Immobility in the FST and the TST can be reduced by non-specific motor activation and increased by sedation [13,14], and venlafaxine has been reported to increase spontaneous locomotor activity in mice at 16 to 64 mg/kg [7], a range that includes two of the three doses used here. No open-field or activity-meter data were collected, so a motor contribution to the reductions in immobility, in particular for VEN 40 mg/kg and for the combination groups, cannot be excluded, and every antidepressant-like effect reported here has to be read with that reservation. Pharmacokinetic measurements were also absent: plasma and brain concentrations of VEN, COD and norBNI were not determined, so pharmacokinetic interactions cannot be excluded. This is particularly pertinent because both VEN and COD undergo CYP2D6-dependent biotransformation in humans, VEN to O-desmethylvenlafaxine [84,85] and COD to morphine, on which its µ-opioid activity depends [86,87]. Murine metabolism involves a distinct Cyp2d gene cluster rather than a single orthologue of human CYP2D6, and interspecies differences in multiplicity and substrate specificity make human pathways difficult to predict from rodent data [88,89]. Competition at blood–brain barrier efflux transporters, including P-glycoprotein, could additionally modify central exposure of both the antidepressant and the opioid components [90,91]. The animals of a treatment group shared a cage, so cage-level effects cannot be separated from treatment effects, and neither the order in which animals were tested within a session nor the position of the cages in the rack was randomised or recorded (Section 4.1). Finally, the four experiments were run as separate cohorts, each with its own saline control, and the control of Experiment 4 was unusually dispersed, so comparisons between treatments that were not tested in the same cohort are indirect.

The computational component carries its own boundaries, and they are worth stating precisely because docking is easily over-read. Docking predicts a binding geometry and ranks it with an empirical function; it does not measure affinity, and the score should not be converted into a predicted dissociation constant. The receptors were held rigid, so side-chain rearrangement on binding was not modelled, and each structure represents a single conformational state: the transporters were docked in the outward-open state and each opioid receptor in the state of its template, which favours ligands that stabilise those states. Four of the eight structures carry a thermostabilising mutation set or a fusion partner, which was removed before docking. Most importantly, the calculations were performed on isolated human proteins, while the behaviour was measured in intact mice, so no docking result can speak to brain exposure, metabolite formation or receptor occupancy in vivo. The structural data support the target assignment on which the combination rests; they do not demonstrate that the predicted complexes form at the doses used here.

Finally, statistical power was adequate for large effects but limited for intermediate ones. The borderline reduction with norBNI + VEN (*p* = 0.091, observed power 0.36) means that a genuine contribution of the binary regimen cannot be excluded, and the non-significant difference between the triple and binary combinations should not be interpreted as evidence of equivalence. Replication in larger cohorts (approximately 16 animals per group for the triple-combination contrast and approximately 35 for the binary contrast) would be required to resolve the quantitative contribution of each component.

### 3.7. Future Directions

Future work should test this combination in chronic stress models and across behavioural domains relevant to treatment-resistant depression, including anhedonia, cognitive dysfunction and motivational impairment. The most informative next experiment is a complete factorial design within a single cohort, comprising saline, each of the three drugs alone, the three binary combinations and the triple combination, since only such a design can establish whether codeine adds anything to κ blockade combined with an SNRI and can avoid the cross-cohort comparisons that limit the present study. Formal isobolographic or response-surface designs [74,75] are then required to determine whether the interaction is supra-additive. Concurrent locomotor controls, separate scoring of active behaviours, neurochemical measurements, receptor-expression profiling and determination of plasma and brain concentrations will clarify the relative contribution of monoaminergic, µ-opioid and κ-opioid mechanisms and will separate motor from affective components. Substituting a clinically usable κ antagonist for norBNI and evaluating both sexes, more than one strain and longer treatment durations, are necessary steps before this multi-target strategy can be considered translationally relevant.

## 4. Materials and Methods

### 4.1. Animals and Ethical Statement

Experiments were conducted on adult male NMRI Swiss albino mice (25–30 g body weight, 8–10 weeks old) supplied to “Carol Davila” University of Medicine and Pharmacy by the National Institute for Medical-Military Research and Development “Cantacuzino” (Bucharest, Romania). Only male animals were used in this initial pharmacological screening in order to minimise behavioural variability related to the oestrous cycle; the inclusion of female mice is planned for subsequent studies. Animals were of conventional microbiological status. Mice with lesions of the tail were not enrolled in the tail suspension experiments; this criterion was set a priori and was applied before allocation.

Animals were group-housed (at most 12 per cage) in Plexiglas cages of two sizes (43 × 33 × 19.5 cm and 36.5 × 20 × 15 cm) with wood-shaving bedding and without environmental enrichment, under controlled conditions (21–24 °C, 45–60% relative humidity, 12 h light/dark cycle, and lights on at 07:00) and acclimatised to the experimental room for at least three days before testing. Food and water were available ad libitum until 1 h before testing. All mice were experimentally naïve, used only once and randomly assigned to treatment groups using a computer-generated randomisation sequence. Behavioural testing was performed between 09:00 and 14:00 to minimise circadian variability. A total of 277 animals were used (85, 60, 60 and 72 in Experiments 1–4, respectively). The animals of a treatment group were housed together from allocation, 2 to 3 days before testing. The order in which individual animals were tested within a session and the position of the cages in the rack were not randomised or recorded.

All procedures complied with EU Directive 2010/63/EU and Romanian Law no. 43/2014 on the protection of animals used for scientific purposes and were approved by the Institutional Ethics Committee for Scientific Research of the Faculty of Medicine, “Carol Davila” University of Medicine and Pharmacy (approval no. 34658, 21 November 2024). The study was additionally authorised as project no. 22 of 24 December 2024 by the Bucharest Sanitary Veterinary and Food Safety Directorate, the competent national authority under Law no. 43/2014. Animals were monitored for general activity, posture, locomotion, grooming, feeding and drinking and for any visible sign of pain or distress throughout the experimental period, including the 24 h after norBNI or vehicle administration, and more closely after each injection; any such sign was the criterion for removal from the experiment, and no animal met it. No adverse effects were observed. All animals were euthanised within 24 h after testing with the inhalational general anaesthetic isoflurane.

Group sizes were justified by an a priori power analysis (Section 4.6), animals were allocated to treatment by a computer-generated randomisation sequence, outcome assessment was blinded, the only criterion applied before allocation was the non-enrolment of animals with tail lesions in the tail suspension experiments, and no animal or data point was excluded from any analysis after allocation.

### 4.2. Drugs and Treatments

VEN hydrochloride (Sigma-Aldrich, St. Louis, MO, USA; cat. no. V7264), AMI hydrochloride (Sigma-Aldrich; cat. no. A8404), COD phosphate (Faculty of Pharmacy, “Carol Davila” University of Medicine and Pharmacy, Bucharest, Romania; pharmaceutical grade) and norBNI dihydrochloride (nor-binaltorphimine dihydrochloride, CAS 113158-34-2, PubChem CID 11957626; Tocris Bioscience, Bristol, UK; cat. no. 0347) were used. All doses are expressed as the salt. Compounds were dissolved in sterile physiological saline (0.9% NaCl), except COD phosphate, which was dissolved in distilled water. Fresh solutions were prepared immediately before each test.

All injections were intraperitoneal (i.p.) in a constant total volume of 10 mL/kg. In Experiments 3 and 4, the agents were injected at separate sites: each mouse received two i.p. injections of 5 mL/kg, one in each hemiabdomen, with each substance consistently assigned to the same hemiabdomen throughout an experiment. Treatment timing was as follows: VEN, COD and saline vehicle 60 min before testing; AMI 20 mg/kg 30 min before testing, in line with its established rodent pharmacokinetics; and norBNI 10 mg/kg 24 h before testing to ensure full and selective κ-opioid receptor blockade consistent with the slow onset and prolonged action of this antagonist [39,40,41]. The same operator prepared and administered all solutions, and the experimenter who scored behaviour was blind to the treatment allocation. All experiments used a parallel-group design, with each group beginning and finishing at approximately the same time of day. Both the total injected volume and the number of injection events were matched within each experiment. In Experiments 1 and 2, every animal received a single injection of 10 mL/kg in the left hemiabdomen. In Experiments 3 and 4, every animal received two injections of 5 mL/kg, one in each hemiabdomen, the vehicle replacing the missing agent in single-agent groups, and in Experiment 4, the groups not treated with norBNI received the corresponding vehicle at the same 24 h time point.

Dose selection. Doses were chosen from published rodent pharmacology rather than from preliminary experiments of our own. For VEN, the 10 to 40 mg/kg range brackets the doses that are active in the mouse forced swim test, where 8 to 64 mg/kg reduce immobility and where serotonin reuptake inhibition predominates at the lower end of the range while noradrenaline reuptake inhibition is progressively recruited at the higher end [7,8]; doses within this range are also those at which the antidepressant-like effect of VEN has been shown to require µ-opioid receptors [12] and an intact opioid system [25]. The same source reports increased spontaneous locomotor activity in mice at 16 to 64 mg/kg [7], which is the principal reason why a motor contribution cannot be excluded for VEN 20 and 40 mg/kg in the present study, although no locomotor change accompanied the antidepressant-like effect of VEN in other reports [11,12]. For COD, the geometric progression of 10, 20 and 40 mg/kg was selected to provide increasing µ-opioid receptor engagement above the sub-effective doses that combine with SSRIs in the mouse TST without affecting motor behaviour [54], so that potentiation and attenuation of the VEN effect would both have been detectable. For norBNI, a single dose of 10 mg/kg given 24 h before testing was used because this dose and this interval produce complete and selective κ-opioid receptor blockade in mice and are the standard regimen in the behavioural literature on κ antagonism [25,39,41]; because the blockade persists for weeks [39,40,41,42], dose escalation within an acute experiment would add nothing. All doses are expressed as the salt and were not adjusted for differences in systemic exposure between compounds, a limitation stated in Section 3.6.

### 4.3. Experimental Design

#### 4.3.1. Experiment 1: Forced Swim Test, Venlafaxine Dose–Response

Five groups of *n* = 17 mice received (a) saline vehicle (negative control); (b) AMI 20 mg/kg (positive control); (c) VEN 10 mg/kg; (d) VEN 20 mg/kg; or (e) VEN 40 mg/kg.

#### 4.3.2. Experiment 2: Tail Suspension Test, Venlafaxine Dose–Response

Five groups of *n* = 12 mice received the same treatments as in Experiment 1 and were tested in the TST 60 min later (30 min for AMI).

#### 4.3.3. Experiment 3: Tail Suspension Test, VEN 20 mg/kg Plus Codeine in Geometric Progression

Six groups of *n* = 10 mice received (a) saline; (b) AMI 20 mg/kg; (c) VEN 20 mg/kg; (d) VEN 20 mg/kg + COD 10 mg/kg; (e) VEN 20 mg/kg + COD 20 mg/kg; or (f) VEN 20 mg/kg + COD 40 mg/kg. VEN and COD were co-administered 60 min before testing.

#### 4.3.4. Experiment 4: Tail Suspension Test, Triple Combination

Six groups of *n* = 12 mice received (a) saline; (b) AMI 20 mg/kg; (c) norBNI 10 mg/kg alone; (d) norBNI 10 mg/kg + VEN 20 mg/kg; (e) norBNI 10 mg/kg + VEN 20 mg/kg + COD 20 mg/kg; or (f) COD 20 mg/kg alone. norBNI was injected 24 h before the TST, VEN and COD 60 min before, and AMI 30 min before. This design is deliberately incomplete as a factorial: VEN 20 mg/kg alone and VEN 20 mg/kg + COD 20 mg/kg were not repeated in this cohort because both had been characterised in Experiment 3, and adding them would have required two further groups of twelve animals for contrasts that were already available, which the reduction principle of the 3Rs does not favour. The incremental contribution of COD in the presence of norBNI is therefore inferred from the binary versus triple comparison within Experiment 4, and any comparison with VEN monotherapy is made across cohorts.

### 4.4. Forced Swim Test

The FST followed a one-session murine protocol modified from Porsolt and colleagues [60,61]. Mice were placed individually in transparent Plexiglas cylinders (height 24 cm, diameter 13 cm) filled with water to a depth of 10 cm at 26 ± 2 °C. Each session lasted 6 min, and total immobility was scored over the last 4 min, the first 2 min serving as a stabilisation period. A mouse was considered immobile when it ceased all active escape attempts and made only those movements necessary to keep its head above water. Sessions were video-recorded (GoPro cameras) and scored offline by a trained observer blind to treatment allocation. The scorer measured active movements in seconds and decimals, and immobility time was obtained by subtracting this value from 240 s. After each trial, the mice were dried in pre-warmed cages.

### 4.5. Tail Suspension Test

The TST followed the procedure of Steru and colleagues [62] with strain-specific modifications [92]. Each animal was suspended by the distal portion of its tail using a 15 cm strip of 1.5 cm wide medical adhesive tape attached to a horizontal metal bar 20 cm above the floor. To prevent tail climbing, a recognised confound in C57BL/6 and NMRI mice [92,93], a rigid plastic tube 3 cm long was placed around the base of the tail before suspension. Sessions lasted 6 min and were video-recorded in full; total immobility was scored offline by an experimenter blind to treatment allocation. Immobility was defined as the absence of all active movements other than those required for respiration. Active movements were measured in seconds and decimals, and immobility time was obtained by subtracting this value from 360 s.

### 4.6. Statistical Analysis

All analyses were planned a priori and performed in GraphPad Prism 10 (GraphPad Software, San Diego, CA, USA), and JASP 0.18.3 (University of Amsterdam, Amsterdam, The Netherlands). Data are presented as the mean ± standard deviation (SD) and as the median with the interquartile range (IQR).

Normality was assessed primarily with the Shapiro–Wilk test, with the Kolmogorov–Smirnov test as a supplementary check; homogeneity of variances was evaluated with Levene’s and Bartlett’s tests. Skewness and kurtosis were computed as descriptive indicators of distribution shape. Normality was satisfied in all groups of Experiment 2 and violated in at least one group of Experiments 1, 3 and 4; variance homogeneity by Levene’s test was satisfied in Experiments 2 and 3 and violated in Experiments 1 and 4 (complete assumption testing in Supplementary Table S1).

Group differences were analysed by one-way ANOVA followed by Dunnett’s test for comparisons versus the saline control (the primary pre-specified analysis) and Tukey’s HSD for all pairwise comparisons. Because assumptions were violated in three of the four experiments, two pre-specified sensitivity analyses were applied throughout: Welch’s heteroscedasticity-robust ANOVA, and Kruskal–Wallis testing followed by Dunn’s post hoc comparisons with Bonferroni correction. All three analytical routes are reported for every experiment so that the reader can judge the robustness of each conclusion.

Effect sizes were quantified as η^2^ and ω^2^ for omnibus effects, ε^2^ for Kruskal–Wallis, and Cohen’s d, Hedges’ g and Cliff’s δ for pairwise contrasts. Hedges’ g is reported with 95% confidence intervals; Cliff’s δ magnitudes follow conventional thresholds (negligible < 0.147, small < 0.33, medium < 0.474, large ≥ 0.474). Percentage change from the control is reported with bias-corrected bootstrap 95% confidence intervals (20,000 resamples, seed fixed for reproducibility). Bayes factors (*BF*_10_) were computed for pairwise contrasts using the Jeffreys–Zellner–Siow prior (Cauchy scale *r* = 0.707) and interpreted with conventional benchmarks. Monotonic dose–response relationships were tested with Spearman rank correlation across ordered dose levels. Planned contrasts involving the triple combination were additionally corrected with the Holm procedure. Statistical significance was defined as a two-tailed *p*-value < 0.05.

For Experiment 4, in which the control group showed high dispersion, three pre-specified sensitivity analyses were performed: symmetric 10% winsorisation, logarithmic transformation and outlier screening by the Tukey 1.5 × IQR criterion. No animals or data points were excluded from any analysis.

The dissociation between the two assays was tested with a linear model on control-standardised immobility scores from Experiments 1 and 2, with the test (FST and TST) as a between-subjects factor, venlafaxine dose (10, 20 and 40 mg/kg) as a continuous predictor and the test × dose interaction, which assesses whether the slope of the dose–response relationship differs between the two assays. Each score was standardised against the saline control of its own experiment as z = (x − control mean)/control SD, so that the two assays could be compared on a common scale. The analysis included 87 animals (*n* = 17 per dose in Experiment 1 and *n* = 12 per dose in Experiment 2) and used Type II sums of squares. Additivity benchmarks for Experiment 4 were computed from the two components available in that cohort, the binary norBNI + VEN regimen (E1 = 0.494) and COD alone (E2 = 0.296), as simple additivity, E1 + E2 = 0.790, and as Bliss independence, 1 − (1 − E1)(1 − E2) = 0.644, where E denotes the fractional reduction in immobility relative to the saline control of the same experiment.

An a priori power analysis was performed in G*Power 3.1.9.7 (Heinrich-Heine-Universität, Düsseldorf, Germany) for one-way ANOVA with five or six groups, α = 0.05 and power (1 − β) = 0.80. Detection of a large effect (*f* = 0.40) required 80 animals in total for five groups and 86 for six groups. Realised sample sizes (60–85 per experiment) were adequate for the large effects observed, and observed power is reported for each omnibus test and for the key pairwise contrasts in Section 2.5.

### 4.7. Molecular Docking

Structures. Eight experimentally determined human structures were retrieved from the RCSB Protein Data Bank and used without homology modelling. The serotonin transporter (SERT, UniProt P31645) was represented by the outward-open ts3 variant in complex with paroxetine (PDB 5I6X, X-ray, 3.14 Å) [77], the noradrenaline transporter (NET, P23975) by the outward-open state in complex with amitriptyline (8Y93, cryo-electron microscopy, 3.00 Å) [76] and the dopamine transporter (DAT, Q01959) by the outward-open state in complex with cocaine (9EO4, cryo-electron microscopy, 2.66 Å) [94]. The μ-opioid receptor (P35372) was represented in the G_i_-coupled active state bound to PZM21 (8EFO, cryo-electron microscopy, 2.80 Å) [95] and in the nanobody-stabilised inactive state bound to naloxone (9PXU, cryo-electron microscopy, 3.40 Å) [96]. The κ-opioid receptor (P41145) was represented in the inactive state bound to JDTic (4DJH, X-ray, 2.90 Å) [78] and in the G_i_-coupled state bound to nor-binaltorphimine (8VVE, cryo-electron microscopy, 3.30 Å) [79] and the δ-opioid receptor (P41143) by the naltrindole complex (4N6H, X-ray, 1.80 Å) [97]. Candidate structures were screened on resolution and on the presence of a co-crystallised orthosteric ligand and were retained only if the redocking criterion defined below was met. Residue numbering follows the deposited coordinates, which in every case is the human sequence numbering.

Receptor preparation. Only the target polypeptide chain was retained. Crystallisation and expression partners were removed, namely T4 lysozyme (4DJH), apocytochrome b562RIL (4N6H), the 8B6 antibody fragment (5I6X), the heterotrimeric G_i_ protein with scFv16 (8EFO, 8VVE) and nanobody 6 with the anti-Fab module (9PXU), together with all water molecules, lipids, detergents, cryoprotectants and ions. Alternate conformations were reduced to the first conformer, and selenomethionine was reverted to methionine. Hydrogen atoms were added at pH 7.4 with PDB2PQR 3.7.1 using the AMBER force field, with residue-specific pKa values predicted by PROPKA 3.5 [98,99]. Non-polar hydrogens were then merged, AutoDock4 atom types were assigned, and each receptor was written as a rigid PDBQT file.

Ligands. Venlafaxine (PubChem CID 5656), codeine (CID 5284371), amitriptyline (CID 2160), morphine (CID 5288826) and nor-binaltorphimine (CID 5480230) were built from their isomeric SMILES strings. Aliphatic amines were assigned their physiological protonation state, which gives monocations for venlafaxine, codeine, amitriptyline and morphine and a dication for nor-binaltorphimine, whose pyrrole nitrogen was left neutral. Three-dimensional coordinates were generated with RDKit 2026.03 using the ETKDGv3 algorithm with a fixed random seed and relaxed with the MMFF94s force field, and torsion trees were assigned with Meeko 0.8.

Docking. Docking was performed with AutoDock Vina 1.2.5 using the default Vina scoring function [81,82]. Search boxes were centred on the centroid of the co-crystallised orthosteric ligand of each structure, with edges set to the ligand extent plus 10 Å and a minimum edge of 22 Å, which encloses the S1 site of the transporters and the orthosteric message–address pocket of the opioid receptors. Exhaustiveness was set to 32. Every ligand–structure pair was docked in three independent runs with fixed random seeds, and the affinity of the top-ranked mode is reported as the mean ± SD across runs; the pose census used the twenty best-scoring modes of the reference run.

Validation and pose selection. Each co-crystallised ligand was extracted from its structure, assigned bond orders from the PDB Chemical Component Dictionary, protonated by the same rule as the test ligands and re-docked into its own receptor under identical settings. Agreement with the experimental pose was quantified as the heavy-atom root-mean-square deviation of the top-ranked mode, computed in the experimental frame without superposition and minimised over all graph automorphisms of the ligand so that topologically equivalent atoms were not penalised; a threshold of 2.0 Å was set a priori and was met by all eight structures. Because empirical scoring functions rank poses imperfectly, a second, pharmacophore-based criterion was applied to the study compounds: among the twenty retained modes, the best-scoring mode forming the conserved ion pair between the protonated ligand nitrogen and the aspartate of transmembrane helix 3 (≤4.0 Å) was identified and is reported alongside the top-ranked mode. The cut-off was derived from the eight experimental complexes themselves.

Interaction analysis. Residues with at least one heavy atom within 4.0 Å of a ligand heavy atom were recorded as contacts. Hydrogen bonds required a donor–acceptor distance of 3.5 Å or less together with a donor–hydrogen–acceptor angle of at least 120°. Salt bridges were defined as a cationic ligand nitrogen within 4.0 Å of an aspartate or glutamate carboxylate oxygen, aromatic stacking as a ring-centroid separation of 5.5 Å or less and cation–π contacts as a cationic nitrogen within 6.0 Å of an aromatic ring centroid. Opioid-receptor residues are given in Ballesteros–Weinstein notation in addition to their sequence numbers [100].

Scope of the computational analysis. Docking predicts binding geometry and returns a rank-ordered empirical score. The Vina score is not a binding free energy in the thermodynamic sense and was not converted into a predicted dissociation or inhibition constant, so comparisons with experimental affinity are restricted to rank order.

## 5. Conclusions

This preclinical study yields three conclusions.

First, the FST and TST dissociate sharply in their sensitivity to venlafaxine in NMRI mice: a dose–response effect was present in the TST and entirely absent in the FST, indicating that under the conditions of this study, the TST was more sensitive to venlafaxine than the FST in this strain; a single compound does not support a general ranking of the two assays.

Second, codeine added to venlafaxine 20 mg/kg does not potentiate the SNRI’s antidepressant-like effect at any of three doses spanning an eightfold range, in contrast to the well-documented supra-additivity of codeine with SSRIs; µ-opioid agonism added to a submaximal SNRI dose did not extend its effect under these conditions, and the cooperative interaction described for codeine and SSRIs appears to be specifically serotonergic.

Third, the triple combination of venlafaxine, codeine and nor-binaltorphimine reduced tail suspension immobility by 65.7% relative to saline (*p* = 0.013), an effect that survived winsorisation and heteroscedasticity-robust analysis and that was similar in magnitude to the effect of amitriptyline, whereas none of the single treatments tested in the same cohort reached significance. This pattern is compatible with sustained κ-opioid blockade creating a permissive context for monoaminergic and µ-opioid facilitation, but it does not demonstrate a cooperative interaction between all three components: the triple combination did not differ significantly from nor-binaltorphimine plus venlafaxine, the contribution of codeine therefore remains undetermined, and the observed effect lies within the range predicted by additivity. The non-significant results obtained for the single treatments were obtained in an underpowered design and do not establish the absence of an effect.

The structural analysis is consistent with the pharmacological premise of the design. Docking against eight human structures was consistent with the site preference of each component, placing venlafaxine at the S1 site of the monoamine transporters rather than at the opioid receptors, codeine at the μ-opioid orthosteric pocket in preference to the κ and δ subtypes and nor-binaltorphimine at the κ receptor through a bivalent binding mode that neither of the other two subtypes can support. The three agents are therefore predicted to occupy separable molecular sites rather than to compete at a shared one. That is a necessary condition for an interaction arising downstream of target binding, but docking cannot show that such an interaction occurs.

These findings justify further, formally designed evaluation of multimodal strategies that integrate monoaminergic and opioid mechanisms while indicating clearly what must come next: isobolographic confirmation, chronic stress models, both sexes, concurrent locomotor and pharmacokinetic measurement and substitution of nor-binaltorphimine by a κ-opioid antagonist compatible with human use, paired with a μ-opioid modulator of lower abuse liability. The structural data presented here identify the sites that such a regimen would engage; whether it engages them at therapeutic exposures is a separate question that docking cannot answer.

## Figures and Tables

**Table 1:** Immobility time, effect sizes and post hoc comparisons across the four experimental series.

Treatment Group	*n*	Mean ± SD (s)	Median [IQR] (s)	Δ vs. Control, % (95% CI)	Hedges’ g (95% CI)	Cliff’s δ	*p* (Dunnett)
***Experiment 1—FST***							
Saline (control)	17	198.98 ± 22.79	199.97 [184.59–219.59]	—	—	—	—
AMI 20	17	71.03 ± 38.68	68.39 [44.00–97.83]	−64.3 (−73.4 to −55.0)	−3.94 (−5.11 to −2.76)	−1.00	<0.001
VEN 10	17	169.34 ± 58.57	175.50 [136.67–218.53]	−14.9 (−29.7 to −0.9)	−0.65 (−1.34 to 0.04)	−0.24	0.188
VEN 20	17	190.36 ± 38.27	200.03 [160.89–219.09]	−4.3 (−14.7 to 5.8)	−0.27 (−0.94 to 0.41)	−0.07	0.951
VEN 40	17	171.89 ± 59.26	183.19 [123.82–222.62]	−13.6 (−28.2 to 0.7)	−0.59 (−1.28 to 0.10)	−0.20	0.254
***Experiment 2—TST***							
Saline (control)	12	161.03 ± 18.21	168.63 [145.40–172.19]	—	—	—	—
AMI 20	12	45.93 ± 29.87	45.09 [22.39–62.86]	−71.5 (−81.2 to −60.9)	−4.49 (−6.04 to −2.94)	−1.00	<0.001
VEN 10	12	123.23 ± 43.25	118.47 [109.53–137.50]	−23.5 (−38.8 to −7.9)	−1.10 (−1.96 to −0.24)	−0.64	0.045
VEN 20	12	102.27 ± 46.62	100.87 [61.63–139.04]	−36.5 (−52.3 to −20.0)	−1.60 (−2.53 to −0.67)	−0.78	<0.001
VEN 40	12	54.42 ± 35.85	48.08 [31.46–72.77]	−66.2 (−77.9 to −53.7)	−3.62 (−4.96 to −2.28)	−1.00	<0.001
***Experiment 3—TST***							
Saline (control)	10	118.80 ± 56.49	107.50 [82.00–137.00]	—	—	—	—
AMI 20	10	30.40 ± 42.96	15.50 [9.00–26.75]	−74.4 (−91.1 to −46.6)	−1.69 (−2.72 to −0.65)	−0.82	<0.001
VEN 20	10	58.90 ± 34.87	47.50 [35.25–86.25]	−50.4 (−69.0 to −24.4)	−1.22 (−2.19 to −0.26)	−0.68	0.013
VEN 20 + COD 10	10	77.00 ± 48.03	66.00 [33.50–116.00]	−35.2 (−61.1 to −1.1)	−0.76 (−1.67 to 0.15)	−0.38	0.129
VEN 20 + COD 20	10	78.20 ± 30.74	78.50 [60.75–95.25]	−34.2 (−54.9 to −6.7)	−0.86 (−1.78 to 0.06)	−0.52	0.146
VEN 20 + COD 40	10	76.20 ± 40.11	70.50 [47.25–113.50]	−35.9 (−59.0 to −4.9)	−0.83 (−1.75 to 0.08)	−0.48	0.118
***Experiment 4—TST***							
Saline (control)	12	127.08 ± 104.88	103.50 [45.75–183.00]	—	—	—	—
AMI 20	12	31.50 ± 26.49	26.00 [8.50–53.50]	−75.2 (−87.6 to −52.1)	−1.21 (−2.08 to −0.33)	−0.57	0.004
norBNI 10	12	113.00 ± 44.76	110.50 [71.25–145.25]	−11.1 (−43.3 to 57.8)	−0.17 (−0.97 to 0.63)	+0.07	0.980
COD 20	12	89.50 ± 80.51	56.00 [49.25–120.75]	−29.6 (−64.8 to 40.3)	−0.39 (−1.20 to 0.42)	−0.15	0.501
norBNI 10 + VEN 20	12	64.25 ± 69.60	54.00 [9.00–77.00]	−49.4 (−77.5 to 5.4)	−0.68 (−1.51 to 0.14)	−0.34	0.091
norBNI 10 + VEN 20 + COD 20	12	43.58 ± 36.43	37.50 [23.50–54.00]	−65.7 (−82.4 to −33.2)	−1.03 (−1.88 to −0.17)	−0.53	0.013

**Table 2:** Summary of antidepressant-like effects and pharmacodynamic interpretation across the four experimental series.

Experiment	Treatment	Δ Immobility (%)	Effect Size Class	Significant vs. Control	Pharmacodynamic Interpretation
1—FST	VEN 10 mg/kg	−14.9	Medium	No	No antidepressant-like activity detectable
1—FST	VEN 20 mg/kg	−4.3	Negligible	No	No antidepressant-like activity detectable
1—FST	VEN 40 mg/kg	−13.6	Medium	No	No antidepressant-like activity detectable; no dose–response trend
2—TST	VEN 10 mg/kg	−23.5	Large	Borderline	Low-dose SNRI activity detectable under Dunnett correction only
2—TST	VEN 20 mg/kg	−36.5	Large	Yes	Submaximal, reproducible antidepressant-like effect
2—TST	VEN 40 mg/kg	−66.2	Very large	Yes	Maximal effect; similar magnitude to AMI, no significant difference detected
3—TST	VEN 20 + COD 10 mg/kg	−35.2	Medium	No	No potentiation of VEN; numerical attenuation
3—TST	VEN 20 + COD 20 mg/kg	−34.2	Large	No	No potentiation of VEN; numerical attenuation
3—TST	VEN 20 + COD 40 mg/kg	−35.9	Large	No	No potentiation of VEN; flat across an eightfold dose range
4—TST	norBNI 10 mg/kg	−11.1	Negligible	No	No significant effect of κ blockade alone
4—TST	COD 20 mg/kg	−29.6	Small	No	No significant effect of µ agonism alone
4—TST	norBNI 10 + VEN 20 mg/kg	−49.4	Medium	No (*p* = 0.091)	Binary regimen underpowered; contribution cannot be excluded
4—TST	norBNI 10 + VEN 20 + COD 20 mg/kg	−65.7	Large	Yes	Significant effect vs. saline; not significantly different from norBNI + VEN or from AMI

Effect-size class refers to Hedges’ g versus the saline control of the same experiment (small ≥ 0.2, medium ≥ 0.5, large ≥ 0.8 and very large ≥ 1.3). “Borderline” denotes significance under Dunnett correction but not under Tukey or Dunn–Bonferroni correction. AMI, amitriptyline; COD, codeine; FST, forced swim test; norBNI, nor-binaltorphimine; SNRI, serotonin–noradrenaline reuptake inhibitor; TST, tail suspension test; VEN, venlafaxine.

**Table 3:** Sensitivity analyses for Experiment 4. Values in the treatment columns are *p* values versus the saline control.

Analysis	Omnibus Test	norBNI 10	COD 20	norBNI + VEN 20	norBNI + VEN 20 + COD 20	AMI 20
Primary (one-way ANOVA + Dunnett)	*F*(5, 66) = 3.99, *p* = 0.003	0.980	0.501	0.091	0.013	0.004
Welch heteroscedasticity-robust	*F*(5, 29.9) = 7.13, *p* < 0.001	—	—	—	—	—
Kruskal–Wallis + Dunn–Bonferroni	*H*(5) = 18.70, *p* = 0.002	1.000	1.000	1.000	0.287	0.112
10% winsorised + Dunnett	*F*(5, 66) = 5.93, *p* < 0.001	0.996	0.295	0.018	0.003	0.001
log(x + 1) transformed + Dunnett	*F*(5, 66) = 2.71, *p* = 0.027	0.947	0.980	0.230	0.222	0.139

**Table 4:** Redocking validation of the eight experimental structures used for molecular docking.

Structure	Co-Crystallised Ligand	RMSD (Å)	Score (kcal mol^−1^)	Anchor Distance (Å)	Anchor Residue
SERT (5I6X)	Paroxetine	0.65	−10.78	3.09	Asp98
NET (8Y93)	Amitriptyline	0.88	−9.33	4.46	Asp75
DAT (9EO4)	Cocaine	0.60	−8.96	3.27	Asp79
μ-OR (active) (8EFO)	PZM21	1.39	−8.17	3.22	Asp149
μ-OR (inactive) (9PXU)	Naloxone	1.04	−8.53	5.71	Asp149
κ-OR (inactive) (4DJH)	JDTic	0.42	−10.78	2.62	Asp138
κ-OR (G_i_-coupled) (8VVE)	nor-Binaltorphimine	0.82	−10.39	3.40	Asp138
δ-OR (4N6H)	Naltrindole	0.17	−11.51	2.87	Asp128

**Table 5:** Docking affinities of the three combination components and the two reference ligands across the eight experimental structures.

Ligand	SERT 5I6X	NET 8Y93	DAT 9EO4	μ-OR 8EFO (Active)	μ-OR 9PXU (Inactive)	κ-OR 4DJH (Inactive)	κ-OR 8VVE (G_i_)	δ-OR 4N6H
Venlafaxine	−7.95	−7.70	−8.55	−6.97	n.a. [−6.87]	−6.97	−5.62	−6.94
Amitriptyline	−7.17	−8.84	−6.33	−6.22	n.a. [−6.50]	−5.87	−5.54	−5.82
Codeine	−7.01	−7.86	−6.24	−7.68	n.a. [−7.82]	−6.70	−5.89	−7.15
Morphine	n.a. [−9.04]	−7.65	−7.36	−7.99	n.a. [−7.96]	−6.69	n.a. [−7.05]	−7.06
nor-Binaltorphimine	n.a. [−3.49]	n.a. [−4.16]	n.a. [−8.51]	−10.14	n.a. [−10.36]	−9.89	−10.20	−9.98

**Table 6:** Interaction inventory of each ligand at its primary target in the best-scoring anchor-consistent pose.

Complex	Score (kcal mol^−1^)	LE	Salt Bridges	Hydrogen Bonds	Aromatic Contacts	Residues ≤ 4 Å
Venlafaxine—SERT	−7.95	0.397	Asp98 (3.86 Å)	Phe335	Tyr176, Tyr95	14
Amitriptyline—NET	−8.84	0.421	Asp75 (3.69 Å); Asp75 (3.91 Å)	–	Phe323, Phe72, Tyr152	13
Codeine—μ-OR (active)	−7.68	0.349	Asp149 (3.59 Å)	Met153	His299, Trp295, Tyr328	11
Morphine—μ-OR (active)	−7.99	0.381	Asp149 (3.10 Å)	Cys219; Asp149	Tyr150	8
nor-Binaltorphimine—κ-OR (G_i_-coupled)	−10.20	0.208	Asp138 (3.26 Å); Glu297 (3.55 Å); Asp138 (3.73 Å)	Asp138	His291, His304, Trp287, Tyr312	17

## Data Availability

The original contributions presented in the study are included in the article; further inquiries can be directed to the first authors.

## Supplementary Materials

The following supporting information can be downloaded at: https://www.mdpi.com/article/10.3390/ijms27219719/s1.

## Abbreviations

The following abbreviations are used in this manuscript:

5-HT	Serotonin (5-hydroxytryptamine)
5-HT_3_	Serotonin 5-HT_3_ receptor
AMI	Amitriptyline
ANOVA	Analysis of variance
BDNF	Brain-derived neurotrophic factor
BF_10_	Bayes factor favouring the alternative hypothesis
CI	Confidence interval
COD	Codeine
CYP2D6	Cytochrome P450 2D6
DAT	Dopamine transporter
FST	Forced swim test
HSD	Honestly significant difference
i.p.	Intraperitoneal
IQR	Interquartile range
JNK1	c-Jun N-terminal kinase 1
LE	Ligand efficiency
MDD	Major depressive disorder
NA	Noradrenaline (norepinephrine)
NET	Noradrenaline transporter
NMRI	Naval Medical Research Institute (mouse strain)
norBNI	Nor-binaltorphimine
PDB	Protein Data Bank
RMSD	Root-mean-square deviation
SD	Standard deviation
SERT	Serotonin transporter
SNRI	Serotonin–noradrenaline reuptake inhibitor
SSRI	Selective serotonin reuptake inhibitor
TRD	Treatment-resistant depression
TST	Tail suspension test
VEN	Venlafaxine
WHO	World Health Organization

## References

[1] World Health Organization (2025). Depressive Disorder (Depression).

[2] GBD 2019 Mental Disorders Collaborators (2022). Global, Regional, and National Burden of 12 Mental Disorders in 204 Countries and Territories, 1990–2019: A Systematic Analysis for the Global Burden of Disease Study 2019. Lancet Psychiatry.

[3] Mathers C.D., Loncar D. (2006). Projections of Global Mortality and Burden of Disease from 2002 to 2030. PLoS Med..

[4] Cambridge O.R., Knight M.J., Mills N., Baune B.T. (2018). The Clinical Relationship between Cognitive Impairment and Psychosocial Functioning in Major Depressive Disorder: A Systematic Review. Psychiatry Res..

[5] Rock P.L., Roiser J.P., Riedel W.J., Blackwell A.D. (2014). Cognitive Impairment in Depression: A Systematic Review and Meta-Analysis. Psychol. Med..

[6] Bymaster F.P., Dreshfield-Ahmad L.J., Threlkeld P.G., Shaw J.L., Thompson L., Nelson D.L., Hemrick-Luecke S.K., Wong D.T. (2001). Comparative Affinity of Duloxetine and Venlafaxine for Serotonin and Norepinephrine Transporters In Vitro and In Vivo, Human Serotonin Receptor Subtypes, and Other Neuronal Receptors. Neuropsychopharmacology.

[7] Redrobe J.P., Bourin M., Colombel M.C., Baker G.B. (1998). Dose-Dependent Noradrenergic and Serotonergic Properties of Venlafaxine in Animal Models Indicative of Antidepressant Activity. Psychopharmacology.

[8] Harvey A.T., Rudolph R.L., Preskorn S.H. (2000). Evidence of the Dual Mechanisms of Action of Venlafaxine. Arch. Gen. Psychiatry.

[9] Holliday S.M., Benfield P. (1995). Venlafaxine. A Review of Its Pharmacology and Therapeutic Potential in Depression. Drugs.

[10] Muth E.A., Haskins J.T., Moyer J.A., Husbands G.E., Nielsen S.T., Sigg E.B. (1986). Antidepressant Biochemical Profile of the Novel Bicyclic Compound Wy-45,030, an Ethyl Cyclohexanol Derivative. Biochem. Pharmacol..

[11] Mitchell H.A., Ahern T.H., Liles L.C., Javors M.A., Weinshenker D. (2006). The Effects of Norepinephrine Transporter Inactivation on Locomotor Activity in Mice. Biol. Psychiatry.

[12] Ide S., Fujiwara S., Fujiwara M., Sora I., Ikeda K., Minami M., Uhl G.R., Ishihara K. (2010). Antidepressant-like Effect of Venlafaxine Is Abolished in Mu-Opioid Receptor-Knockout Mice. J. Pharmacol. Sci..

[13] Slattery D.A., Cryan J.F. (2012). Using the Rat Forced Swim Test to Assess Antidepressant-like Activity in Rodents. Nat. Protoc..

[14] Porsolt R.D., Anton G., Blavet N., Jalfre M. (1978). Behavioural Despair in Rats: A New Model Sensitive to Antidepressant Treatments. Eur. J. Pharmacol..

[15] Rush A.J., Trivedi M.H., Wisniewski S.R., Nierenberg A.A., Stewart J.W., Warden D., Niederehe G., Thase M.E., Lavori P.W., Lebowitz B.D. (2006). Acute and Longer-Term Outcomes in Depressed Outpatients Requiring One or Several Treatment Steps: A STAR*D Report. Am. J. Psychiatry.

[16] McIntyre R.S., Alsuwaidan M., Baune B.T., Berk M., Demyttenaere K., Goldberg J.F., Gorwood P., Ho R., Kasper S., Kennedy S.H. (2023). Treatment-Resistant Depression: Definition, Prevalence, Detection, Management, and Investigational Interventions. World Psychiatry.

[17] Sforzini L., Worrell C., Kose M., Anderson I.M., Aouizerate B., Arolt V., Bauer M., Baune B.T., Blier P., Cleare A.J. (2022). A Delphi-Method-Based Consensus Guideline for Definition of Treatment-Resistant Depression for Clinical Trials. Mol. Psychiatry.

[18] Zhdanava M., Pilon D., Ghelerter I., Chow W., Joshi K., Lefebvre P., Sheehan J.J. (2021). The Prevalence and National Burden of Treatment-Resistant Depression and Major Depressive Disorder in the United States. J. Clin. Psychiatry.

[19] Harmer C.J., Duman R.S., Cowen P.J. (2017). How Do Antidepressants Work? New Perspectives for Refining Future Treatment Approaches. Lancet Psychiatry.

[20] Taylor M.J., Freemantle N., Geddes J.R., Bhagwagar Z. (2006). Early Onset of Selective Serotonin Reuptake Inhibitor Antidepressant Action: Systematic Review and Meta-Analysis. Arch. Gen. Psychiatry.

[21] Markowitz J.S., Patrick K.S. (1998). Venlafaxine-Tramadol Similarities. Med. Hypotheses.

[22] Mignat C., Wille U., Ziegler A. (1995). Affinity Profiles of Morphine, Codeine, Dihydrocodeine and Their Glucuronides at Opioid Receptor Subtypes. Life Sci..

[23] Portoghese P.S., Lipkowski A.W., Takemori A.E. (1987). Binaltorphimine and Nor-Binaltorphimine, Potent and Selective Kappa-Opioid Receptor Antagonists. Life Sci..

[24] Takemori A.E., Ho B.Y., Naeseth J.S., Portoghese P.S. (1988). Nor-Binaltorphimine, a Highly Selective Kappa-Opioid Antagonist in Analgesic and Receptor Binding Assays. J. Pharmacol. Exp. Ther..

[25] Berrocoso E., Rojas-Corrales M.O., Mico J.A. (2004). Non-Selective Opioid Receptor Antagonism of the Antidepressant-like Effect of Venlafaxine in the Forced Swimming Test in Mice. Neurosci. Lett..

[26] Schreiber S., Backer M.M., Pick C.G. (1999). The Antinociceptive Effect of Venlafaxine in Mice Is Mediated through Opioid and Adrenergic Mechanisms. Neurosci. Lett..

[27] Schreiber S., Bleich A., Pick C.G. (2002). Venlafaxine and Mirtazapine: Different Mechanisms of Antidepressant Action, Common Opioid-Mediated Antinociceptive Effects-A Possible Opioid Involvement in Severe Depression?. J. Mol. Neurosci..

[28] Lungu C., Marin R.-C., Costescu M., Zugravu A., Paunescu H., Ghita C.I., Coman O.A. (2026). Venlafaxine as Monotherapy and in Combination Regimens in Acute Rodent Nociception Experimental Models: A Review. Int. J. Mol. Sci..

[29] Schreiber S., Keidan L., Pick C.G. (2023). Treatment-Resistant Depression (TRD): Is the Opioid System Involved?. Int. J. Mol. Sci..

[30] Lutz P.-E., Kieffer B.L. (2013). Opioid Receptors: Distinct Roles in Mood Disorders. Trends Neurosci..

[31] Pfeiffer A., Brantl V., Herz A., Emrich H.M. (1986). Psychotomimesis Mediated by Kappa Opiate Receptors. Science.

[32] Mague S.D., Pliakas A.M., Todtenkopf M.S., Tomasiewicz H.C., Zhang Y., Stevens W.C., Jones R.M., Portoghese P.S., Carlezon W.A. (2003). Antidepressant-like Effects of Kappa-Opioid Receptor Antagonists in the Forced Swim Test in Rats. J. Pharmacol. Exp. Ther..

[33] Land B.B., Bruchas M.R., Lemos J.C., Xu M., Melief E.J., Chavkin C. (2008). The Dysphoric Component of Stress Is Encoded by Activation of the Dynorphin Kappa-Opioid System. J. Neurosci..

[34] Knoll A.T., Muschamp J.W., Daws S.E., Ferguson D., Dietz D.M., Meloni E.G., Carroll F.I., Nestler E.J., Konradi C., Carlezon W.A. (2011). Kappa Opioid Receptor Signaling in the Basolateral Amygdala Regulates Conditioned Fear and Anxiety in Rats. Biol. Psychiatry.

[35] Bruchas M.R., Land B.B., Chavkin C. (2010). The Dynorphin/Kappa Opioid System as a Modulator of Stress-Induced and Pro-Addictive Behaviors. Brain Res..

[36] Knoll A.T., Carlezon W.A. (2010). Dynorphin, Stress, and Depression. Brain Res..

[37] Karkhanis A.N., Huggins K.N., Rose J.H., Jones S.R. (2016). Switch from Excitatory to Inhibitory Actions of Ethanol on Dopamine Levels after Chronic Exposure: Role of Kappa Opioid Receptors. Neuropharmacology.

[38] Ehrich J.M., Messinger D.I., Knakal C.R., Kuhar J.R., Schattauer S.S., Bruchas M.R., Zweifel L.S., Kieffer B.L., Phillips P.E.M., Chavkin C. (2015). Kappa Opioid Receptor-Induced Aversion Requires p38 MAPK Activation in VTA Dopamine Neurons. J. Neurosci..

[39] Horan P., Taylor J., Yamamura H.I., Porreca F. (1992). Extremely Long-Lasting Antagonistic Actions of Nor-Binaltorphimine (Nor-BNI) in the Mouse Tail-Flick Test. J. Pharmacol. Exp. Ther..

[40] Knoll A.T., Meloni E.G., Thomas J.B., Carroll F.I., Carlezon W.A. (2007). Anxiolytic-like Effects of Kappa-Opioid Receptor Antagonists in Models of Unlearned and Learned Fear in Rats. J. Pharmacol. Exp. Ther..

[41] Bruchas M.R., Yang T., Schreiber S., Defino M., Kwan S.C., Li S., Chavkin C. (2007). Long-Acting Kappa Opioid Antagonists Disrupt Receptor Signaling and Produce Noncompetitive Effects by Activating c-Jun N-Terminal Kinase. J. Biol. Chem..

[42] Melief E.J., Miyatake M., Carroll F.I., Beguin C., Carlezon W.A., Cohen B.M., Grimwood S., Mitch C.H., Rorick-Kehn L., Chavkin C. (2011). Duration of Action of a Broad Range of Selective Kappa-Opioid Receptor Antagonists Is Positively Correlated with c-Jun N-Terminal Kinase-1 Activation. Mol. Pharmacol..

[43] Zhang H., Shi Y.-G., Woods J.H., Watson S.J., Ko M.-C. (2007). Central Kappa-Opioid Receptor-Mediated Antidepressant-like Effects of Nor-Binaltorphimine: Behavioral and BDNF mRNA Expression Studies. Eur. J. Pharmacol..

[44] Zivanovic A., Mitic M., Lukic I., Glavonic E., Adzic M., Ivkovic S. (2026). Kappa-Opioid Receptor Antagonism Prolongs the Antidepressant Effects of Ketamine in Adult Mice with Depression-like Behavior Induced by Adolescent Chronic Unpredictable Stress. Int. J. Mol. Sci..

[45] Fava M., Memisoglu A., Thase M.E., Bodkin J.A., Trivedi M.H., de Somer M., Du Y., Leigh-Pemberton R., DiPetrillo L., Silverman B. (2016). Opioid Modulation with Buprenorphine/Samidorphan as Adjunctive Treatment for Inadequate Response to Antidepressants: A Randomized Double-Blind Placebo-Controlled Trial. Am. J. Psychiatry.

[46] Fava M., Thase M.E., Trivedi M.H., Ehrich E., Martin W.F., Memisoglu A., Nangia N., Stanford A.D., Yu M., Pathak S. (2020). Opioid System Modulation with Buprenorphine/Samidorphan Combination for Major Depressive Disorder: Two Randomized Controlled Studies. Mol. Psychiatry.

[47] Krystal A.D., Pizzagalli D.A., Smoski M., Mathew S.J., Nurnberger J., Lisanby S.H., Iosifescu D., Murrough J.W., Yang H., Weiner R.D. (2020). A Randomized Proof-of-Mechanism Trial Applying the ‘Fast-Fail’ Approach to Evaluating Kappa-Opioid Antagonism as a Treatment for Anhedonia. Nat. Med..

[48] Demyttenaere K. (2025). Aticaprant, a Kappa Opioid Receptor Antagonist, and the Recovered ‘Interest and Pleasure’ in the Concept of Major Depressive Disorder. Eur. Arch. Psychiatry Clin. Neurosci..

[49] Fava M., Mazzone E., Freeman M., Flynn M., Judge H., Hoeppner B., Hock R.S., Shui A., Macaluso M., Morrison M.F. (2020). Double-Blind, Placebo-Controlled, Proof-of-Concept Trial of a Kappa-Selective Opioid Receptor Antagonist Augmentation in Treatment-Resistant Depression. Ann. Clin. Psychiatry.

[50] Wong S., Le G.H., Vasudeva S., Teopiz K.M., Phan L., Meshkat S., Kwan A.T.H., Rhee T.G., Ho R., Choi H. (2024). Preclinical and Clinical Efficacy of Kappa Opioid Receptor Antagonists for Depression: A Systematic Review. J. Affect. Disord..

[51] Page S., Mavrikaki M.M., Lintz T., Puttick D., Roberts E., Rosen H., Carroll F.I., Carlezon W.A., Chartoff E.H. (2019). Behavioral Pharmacology of Novel Kappa Opioid Receptor Antagonists in Rats. Int. J. Neuropsychopharmacol..

[52] Carlezon W.A., Krystal A.D. (2016). Kappa-Opioid Antagonists for Psychiatric Disorders: From Bench to Clinical Trials. Depress. Anxiety.

[53] Carroll F.I., Carlezon W.A. (2013). Development of Kappa Opioid Receptor Antagonists. J. Med. Chem..

[54] Berrocoso E., Mico J.A. (2009). Cooperative Opioid and Serotonergic Mechanisms Generate Superior Antidepressant-like Effects in a Mice Model of Depression. Int. J. Neuropsychopharmacol..

[55] Berrocoso E., Ikeda K., Sora I., Uhl G.R., Sanchez-Blazquez P., Mico J.A. (2013). Active Behaviours Produced by Antidepressants and Opioids in the Mouse Tail Suspension Test. Int. J. Neuropsychopharmacol..

[56] Moron J.A., Brockington A., Wise R.A., Rocha B.A., Hope B.T. (2002). Dopamine Uptake through the Norepinephrine Transporter in Brain Regions with Low Levels of the Dopamine Transporter: Evidence from Knock-Out Mouse Lines. J. Neurosci..

[57] Johnson S.W., North R.A. (1992). Opioids Excite Dopamine Neurons by Hyperpolarization of Local Interneurons. J. Neurosci..

[58] Cryan J.F., Mombereau C., Vassout A. (2005). The Tail Suspension Test as a Model for Assessing Antidepressant Activity: Review of Pharmacological and Genetic Studies in Mice. Neurosci. Biobehav. Rev..

[59] Petit-Demouliere B., Chenu F., Bourin M. (2005). Forced Swimming Test in Mice: A Review of Antidepressant Activity. Psychopharmacology.

[60] Porsolt R.D., Le Pichon M., Jalfre M. (1977). Depression: A New Animal Model Sensitive to Antidepressant Treatments. Nature.

[61] Porsolt R.D., Bertin A., Jalfre M. (1977). Behavioral Despair in Mice: A Primary Screening Test for Antidepressants. Arch. Int. Pharmacodyn. Ther..

[62] Steru L., Chermat R., Thierry B., Simon P. (1985). The Tail Suspension Test: A New Method for Screening Antidepressants in Mice. Psychopharmacology.

[63] Commons K.G., Cholanians A.B., Babb J.A., Ehlinger D.G. (2017). The Rodent Forced Swim Test Measures Stress-Coping Strategy, Not Depression-like Behavior. ACS Chem. Neurosci..

[64] Molendijk M.L., de Kloet E.R. (2019). Coping with the Forced Swim Stressor: Current State-of-the-Art. Behav. Brain Res..

[65] Cryan J.F., Holmes A. (2005). The Ascent of Mouse: Advances in Modelling Human Depression and Anxiety. Nat. Rev. Drug Discov..

[66] Ripoll N., David D.J., Dailly E., Hascoet M., Bourin M. (2003). Antidepressant-like Effects in Various Mice Strains in the Tail Suspension Test. Behav. Brain Res..

[67] Bourin M., Chenu F., Ripoll N., David D.J. (2005). A Proposal of Decision Tree to Screen Putative Antidepressants Using Forced Swim and Tail Suspension Tests. Behav. Brain Res..

[68] Ramamoorthy R., Radhakrishnan M., Borah M. (2008). Antidepressant-like Effects of Serotonin Type-3 Antagonist, Ondansetron: An Investigation in Behaviour-Based Rodent Models. Behav. Pharmacol..

[69] Gautam B.K., Jindal A., Dhar A.K., Mahesh R. (2013). Antidepressant-like Activity of 2-(4-Phenylpiperazin-1-yl)-1,8-Naphthyridine-3-Carboxylic Acid (7a), a 5-HT3 Receptor Antagonist in Behaviour Based Rodent Models: Evidence for the Involvement of Serotonergic System. Pharmacol. Biochem. Behav..

[70] Vanderah T.W., Gardell L.R., Burgess S.E., Ibrahim M., Dogrul A., Zhong C.M., Zhang E.T., Malan T.P., Ossipov M.H., Lai J. (2000). Dynorphin Promotes Abnormal Pain and Spinal Opioid Antinociceptive Tolerance. J. Neurosci..

[71] Horner K.A., Keefe K.A. (2006). Regulation of Psychostimulant-Induced Preprodynorphin, c-Fos and Zif/268 Messenger RNA Expression in the Rat Dorsal Striatum by Mu Opioid Receptor Blockade. Eur. J. Pharmacol..

[72] Pan Z.Z. (1998). Mu-Opposing Actions of the Kappa-Opioid Receptor. Trends Pharmacol. Sci..

[73] Tallarida R.J. (2001). Drug Synergism: Its Detection and Applications. J. Pharmacol. Exp. Ther..

[74] Tallarida R.J. (2011). Quantitative Methods for Assessing Drug Synergism. Genes Cancer.

[75] Tallarida R.J. (2012). Revisiting the Isobole and Related Quantitative Methods for Assessing Drug Synergism. J. Pharmacol. Exp. Ther..

[76] Zhang H., Yin Y.-L., Dai A., Zhang T., Zhang C., Wu C., Hu W., He X., Pan B., Jin S. (2024). Dimerization and Antidepressant Recognition at Noradrenaline Transporter. Nature.

[77] Coleman J.A., Green E.M., Gouaux E. (2016). X-ray Structures and Mechanism of the Human Serotonin Transporter. Nature.

[78] Wu H., Wacker D., Mileni M., Katritch V., Han G.W., Vardy E., Liu W., Thompson A.A., Huang X.-P., Carroll F.I. (2012). Structure of the Human Kappa-Opioid Receptor in Complex with JDTic. Nature.

[79] Tyson A.S., Khan S., Motiwala Z., Han G.W., Zhang Z., Ranjbar M., Styrpejko D., Ramos-Gonzalez N., Woo S., Villers K. (2025). Molecular Mechanisms of Inverse Agonism via Kappa-Opioid Receptor-G Protein Complexes. Nat. Chem. Biol..

[80] Tatsumi M., Groshan K., Blakely R.D., Richelson E. (1997). Pharmacological Profile of Antidepressants and Related Compounds at Human Monoamine Transporters. Eur. J. Pharmacol..

[81] Trott O., Olson A.J. (2010). AutoDock Vina: Improving the Speed and Accuracy of Docking with a New Scoring Function, Efficient Optimization, and Multithreading. J. Comput. Chem..

[82] Eberhardt J., Santos-Martins D., Tillack A.F., Forli S. (2021). AutoDock Vina 1.2.0: New Docking Methods, Expanded Force Field, and Python Bindings. J. Chem. Inf. Model..

[83] Kathiramalainathan K., Kaplan H.L., Romach M.K., Busto U.E., Li N.Y., Sawe J., Tyndale R.F., Sellers E.M. (2000). Inhibition of Cytochrome P450 2D6 Modifies Codeine Abuse Liability. J. Clin. Psychopharmacol..

[84] Fukuda T., Yamamoto I., Nishida Y., Zhou Q., Ohno M., Takada K., Azuma J. (1999). Effect of the CYP2D6*10 Genotype on Venlafaxine Pharmacokinetics in Healthy Adult Volunteers. Br. J. Clin. Pharmacol..

[85] Shams M.E.E., Arneth B., Hiemke C., Dragicevic A., Muller M.J., Kaiser R., Lackner K., Hartter S. (2006). CYP2D6 Polymorphism and Clinical Effect of the Antidepressant Venlafaxine. J. Clin. Pharm. Ther..

[86] Desmeules J., Gascon M.P., Dayer P., Magistris M. (1991). Impact of Environmental and Genetic Factors on Codeine Analgesia. Eur. J. Clin. Pharmacol..

[87] Crews K.R., Monte A.A., Huddart R., Caudle K.E., Kharasch E.D., Gaedigk A., Dunnenberger H.M., Leeder J.S., Callaghan J.T., Samer C.F. (2021). Clinical Pharmacogenetics Implementation Consortium Guideline for CYP2D6, OPRM1, and COMT Genotypes and Select Opioid Therapy. Clin. Pharmacol. Ther..

[88] Scheer N., Kapelyukh Y., McEwan J., Beuger V., Stanley L.A., Rode A., Wolf C.R. (2012). Modeling Human Cytochrome P450 2D6 Metabolism and Drug-Drug Interaction by a Novel Panel of Knockout and Humanized Mouse Lines. Mol. Pharmacol..

[89] Martignoni M., Groothuis G.M.M., de Kanter R. (2006). Species Differences between Mouse, Rat, Dog, Monkey and Human CYP-Mediated Drug Metabolism, Inhibition and Induction. Expert Opin. Drug Metab. Toxicol..

[90] O’Brien F.E., Dinan T.G., Griffin B.T., Cryan J.F. (2012). Interactions between Antidepressants and P-Glycoprotein at the Blood-Brain Barrier: Clinical Significance of In Vitro and In Vivo Findings. Br. J. Pharmacol..

[91] Chaves C., Remiao F., Cisternino S., Decleves X. (2017). Opioids and the Blood-Brain Barrier: A Dynamic Interaction with Consequences on Drug Disposition in Brain. Curr. Neuropharmacol..

[92] Can A., Dao D.T., Terrillion C.E., Piantadosi S.C., Bhat S., Gould T.D. (2012). The Tail Suspension Test. J. Vis. Exp..

[93] Mayorga A.J., Lucki I. (2001). Limitations on the Use of the C57BL/6 Mouse in the Tail Suspension Test. Psychopharmacology.

[94] Nielsen J.C., Salomon K., Kalenderoglou I.E., Bargmeyer S., Pape T., Shahsavar A., Loland C.J. (2024). Structure of the Human Dopamine Transporter in Complex with Cocaine. Nature.

[95] Zhuang Y., Wang Y., He B., He X., Zhou X.E., Guo S., Rao Q., Yang J., Liu J., Zhou Q. (2022). Molecular Recognition of Morphine and Fentanyl by the Human μ-Opioid Receptor. Cell.

[96] Khan S., Tyson A.S., Ranjbar M., Zhang Z., Singh J., Han G.W., Gati C. (2025). Structural Snapshots Capture Nucleotide Release at the μ-Opioid Receptor. Nature.

[97] Fenalti G., Giguere P.M., Katritch V., Huang X.-P., Thompson A.A., Cherezov V., Roth B.L., Stevens R.C. (2014). Molecular Control of Delta-Opioid Receptor Signalling. Nature.

[98] Dolinsky T.J., Nielsen J.E., McCammon J.A., Baker N.A. (2004). PDB2PQR: An Automated Pipeline for the Setup of Poisson-Boltzmann Electrostatics Calculations. Nucleic Acids Res..

[99] Olsson M.H.M., Sondergaard C.R., Rostkowski M., Jensen J.H. (2011). PROPKA3: Consistent Treatment of Internal and Surface Residues in Empirical pKa Predictions. J. Chem. Theory Comput..

[100] Ballesteros J.A., Weinstein H. (1995). Integrated Methods for the Construction of Three-Dimensional Models and Computational Probing of Structure-Function Relations in G Protein-Coupled Receptors. Methods Neurosci..

