# Supplementary material for: Antidepressant-like Activity of a Triple Combination of Venlafaxine, Codeine and Nor-Binaltorphimine in the Mouse Tail Suspension Test
Source: Int J Mol Sci. 2026 Oct 29;27(21):9719. doi: 10.3390/ijms27219719 (PMC13763530; doi:10.3390/ijms27219719)
Supplement: Supplementary file 1 [file ijms-27-09719-s001.zip › Supplementary Table S1.pdf]

Suplementary Table S1. Complete descriptive statistics and assumption-testing outputs for all experimental series.

Table S1. Group-level descriptive statistics for the four experimental series. Skewness and kurtosis are reported as Fisher coefficients (0 = perfectly normal). The Shapiro–Wilk test was applied as the primary normality criterion (H_0: data are normally distributed). Levene's test (median-centred) assessed homogeneity of variances across groups within each experiment (H_0: equal variances). Combined conclusions guided the choice between parametric (ANOVA + Tukey/Dunnett) and non-parametric (Kruskal–Wallis + Dunn) inferential analyses.

Experiment 1 — FST, venlafaxine dose-response (n = 17 per group)

Group	n	Mean ± SD	Median (IQR)	Min–Max	Skewness	Kurtosis	Shapiro–Wilk W	Shapiro p-value
Saline (Control)	17	198.98 ± 22.79	200.0 (184.6–219.6)	156.2–229.9	-0.32	-0.95	0.949	0.443
AMI 20 mg/kg	17	71.03 ± 38.68	68.4 (44.0–97.8)	12.1–132.8	-0.06	-1.19	0.950	0.457
VEN 10 mg/kg	17	169.34 ± 58.57	175.5 (136.7–218.5)	33.1–237.1	-0.76	-0.27	0.905	0.084
VEN 20 mg/kg	17	190.36 ± 38.27	200.0 (160.9–219.1)	105.2–236.2	-0.82	-0.44	0.901	0.072
VEN 40 mg/kg	17	171.89 ± 59.26	183.2 (123.8–222.6)	29.2–235.6	-0.81	-0.16	0.892	0.049

Levene's test (median-centred): W = 4.075, p = 0.005. **One-way ANOVA:** F(4,80) = 21.58, p = 4.21e-12, η^2 = 0.519. **Kruskal–Wallis:** H(4) = 34.72, p = 5.3e-07.

Experiment 2 — TST, venlafaxine dose-response (n = 12 per group)

Group	n	Mean ± SD	Median (IQR)	Min–Max	Skewness	Kurtosis	Shapiro–Wilk W	Shapiro p-value
Saline (Control)	12	161.03 ± 18.21	168.6 (145.4–172.2)	128.8–186.5	-0.46	-1.06	0.924	0.318
AMI 20 mg/kg	12	45.93 ± 29.87	45.1 (22.4–62.9)	7.3–98.3	+0.43	-0.90	0.938	0.476
VEN 10 mg/kg	12	123.23 ± 43.25	118.5 (109.5–137.5)	34.9–203.7	-0.06	+0.24	0.943	0.540
VEN 20 mg/kg	12	102.27 ± 46.62	100.9 (61.6–139.0)	43.5–167.4	+0.24	-1.34	0.904	0.178

VEN 40 mg/kg	12	54.42 ± 35.85	48.1 (31.5–72.8)	4.8–123.7	+0.44	-0.66	0.964	0.833

Levene's test (median-centred): W = 1.909, p = 0.122. **One-way ANOVA:** F(4,55) = 21.14, p = 1.34e-10, η^2 = 0.606. **Kruskal–Wallis:** H(4) = 36.28, p = 2.54e-07.

Experiment 3 — TST, VEN 20 mg/kg + codeine geometric series (n = 10 per group)

Group	n	Mean ± SD	Median (IQR)	Min–Max	Skewness	Kurtosis	Shapiro–Wilk W	Shapiro p-value
Saline (Control)	10	118.80 ± 56.49	107.5 (82.0–137.0)	60.0–259.0	+1.56	+1.91	0.818	0.024
AMI 20 mg/kg	10	30.40 ± 42.96	15.5 (9.0–26.8)	0.0–144.0	+2.08	+3.12	0.672	0.000
VEN 20 mg/kg	10	58.90 ± 34.87	47.5 (35.2–86.2)	18.0–123.0	+0.52	-0.96	0.923	0.386
VEN 20 + COD 10 mg/kg	10	77.00 ± 48.03	66.0 (33.5–116.0)	20.0–144.0	+0.27	-1.49	0.891	0.175
VEN 20 + COD 20 mg/kg	10	78.20 ± 30.74	78.5 (60.8–95.2)	18.0–122.0	-0.35	-0.28	0.964	0.834
VEN 20 + COD 40 mg/kg	10	76.20 ± 40.11	70.5 (47.2–113.5)	17.0–134.0	+0.11	-1.28	0.947	0.633

Levene's test (median-centred): W = 0.666, p = 0.651. **One-way ANOVA:** F(5,54) = 4.50, p = 0.00169, η^2 = 0.294. **Kruskal–Wallis:** H(5) = 18.16, p = 0.00275.

Experiment 4 — TST, triple combination (n = 12 per group)

Group	n	Mean ± SD	Median (IQR)	Min–Max	Skewness	Kurtosis	Shapiro–Wilk W	Shapiro p-value
Saline (Control)	12	127.08 ± 104.88	103.5 (45.8–183.0)	0.0–336.0	+0.60	-0.77	0.907	0.197
AMI 20 mg/kg	12	31.50 ± 26.49	26.0 (8.5–53.5)	2.0–78.0	+0.44	-1.15	0.911	0.217
norBNI 10 mg/kg	12	113.00 ± 44.76	110.5 (71.2–145.2)	50.0–180.0	+0.14	-1.32	0.939	0.481
norBNI 10 + VEN 20 mg/kg	12	64.25 ± 69.60	54.0 (9.0–77.0)	0.0–240.0	+1.41	+1.45	0.826	0.019
norBNI 10 + VEN 20 + COD 20 mg/kg	12	43.58 ± 36.43	37.5 (23.5–54.0)	0.0–117.0	+0.71	-0.28	0.915	0.248
COD 20 mg/kg	12	89.50 ± 80.51	56.0 (49.2–120.8)	0.0–278.0	+1.20	+0.52	0.856	0.043

Levene's test (median-centred): W = 3.533, p = 0.007. **One-way ANOVA:** F(5,66) = 3.99, p = 0.00319, η^2 = 0.232. **Kruskal–Wallis:** H(5) = 18.70, p = 0.00219.
