# Supplementary material for: Antidepressant-like Activity of a Triple Combination of Venlafaxine, Codeine and Nor-Binaltorphimine in the Mouse Tail Suspension Test
Source: Int J Mol Sci. 2026 Oct 29;27(21):9719. doi: 10.3390/ijms27219719 (PMC13763530; doi:10.3390/ijms27219719)
Supplement: Supplementary file 1 [file ijms-27-09719-s001.zip › Supplementary Table S2.pdf]

Supplementary Table S2. Complete Tukey, Dunnett and Dunn post-hoc comparison matrices

Table S2. Full post-hoc comparison output for each experiment. The Tukey HSD test reports all pairwise mean differences with family-wise error correction (FWER = 0.05). Dunnett's test reports comparisons of each treatment against the saline control. Dunn's test is the non-parametric counterpart of Tukey, with Bonferroni correction. Adjusted p-values are reported throughout; bold entries indicate $p < 0.05$.

Experiment 1 — FST, venlafaxine dose-response (n = 17 per group)

Tukey HSD — all pairwise comparisons

Comparison	Mean diff	Lower 95% CI	Upper 95% CI	p-value (Tukey)
AMI 20 mg/kg vs. Saline (Control)	+127.94	+84.24	+171.65	< 0.001
AMI 20 mg/kg vs. VEN 10 mg/kg	+98.31	+54.60	+142.02	
AMI 20 mg/kg vs. VEN 20 mg/kg	+119.33	+75.62	+163.03	
AMI 20 mg/kg vs. VEN 40 mg/kg	+100.86	+57.15	+144.57	
Saline (Control) vs. VEN 10 mg/kg	-29.63	-73.34	+14.07	0.33
Saline (Control) vs. VEN 20 mg/kg	-8.62	-52.32	+35.09	0.982
Saline (Control) vs. VEN 40 mg/kg	-27.08	-70.79	+16.63	0.422
VEN 10 mg/kg vs. VEN 20 mg/kg	+21.02	-22.69	+64.72	0.666
VEN 10 mg/kg vs. VEN 40 mg/kg	+2.55	-41.16	+46.26	1
VEN 20 mg/kg vs. VEN 40 mg/kg	-18.47	-62.17	+25.24	0.763

Dunnett's test — vs. Saline control

Comparison	t-statistic	p-value (Dunnett)
AMI 20 mg/kg vs. Saline (Control)	-8.170	< 0.001
VEN 10 mg/kg vs. Saline (Control)	-1.892	0.188
VEN 20 mg/kg vs. Saline (Control)	-0.550	0.951
VEN 40 mg/kg vs. Saline (Control)	-1.729	0.254

Dunn's test — non-parametric pairwise (Bonferroni-adjusted)

Comparison	p-value (Dunn–Bonferroni)
AMI 20 mg/kg vs. Saline (Control)	< 0.001
AMI 20 mg/kg vs. VEN 10 mg/kg	0.000557

AMI 20 mg/kg vs. VEN 20 mg/kg	< 0.001
AMI 20 mg/kg vs. VEN 40 mg/kg	0.000315
Saline (Control) vs. VEN 10 mg/kg	1
Saline (Control) vs. VEN 20 mg/kg	
Saline (Control) vs. VEN 40 mg/kg	
VEN 10 mg/kg vs. VEN 20 mg/kg	
VEN 10 mg/kg vs. VEN 40 mg/kg	
VEN 20 mg/kg vs. VEN 40 mg/kg	

Experiment 2 — TST, venlafaxine dose-response (n = 12 per group)

Tukey HSD — all pairwise comparisons

Comparison	Mean diff	Lower 95% CI	Upper 95% CI	p-value (Tukey)
AMI 20 mg/kg vs. Saline (Control)	+115.10	+73.42	+156.78	< 0.001
AMI 20 mg/kg vs. VEN 10 mg/kg	+77.30	+35.62	+118.98	
AMI 20 mg/kg vs. VEN 20 mg/kg	+56.34	+14.66	+98.03	0.0031
AMI 20 mg/kg vs. VEN 40 mg/kg	+8.49	-33.19	+50.18	0.978
Saline (Control) vs. VEN 10 mg/kg	-37.80	-79.48	+3.89	0.0928
Saline (Control) vs. VEN 20 mg/kg	-58.75	-100.44	-17.07	0.0019
Saline (Control) vs. VEN 40 mg/kg	-106.60	-148.29	-64.92	< 0.001
VEN 10 mg/kg vs. VEN 20 mg/kg	-20.96	-62.64	+20.73	0.619
VEN 10 mg/kg vs. VEN 40 mg/kg	-68.81	-110.49	-27.12	0.0002
VEN 20 mg/kg vs. VEN 40 mg/kg	-47.85	-89.53	-6.17	0.0168

Dunnett's test — vs. Saline control

Comparison	t-statistic	p-value (Dunnett)
AMI 20 mg/kg vs. Saline (Control)	-7.788	< 0.001
VEN 10 mg/kg vs. Saline (Control)	-2.557	0.0451
VEN 20 mg/kg vs. Saline (Control)	-3.975	0.000774
VEN 40 mg/kg vs. Saline (Control)	-7.213	< 0.001

Dunn's test — non-parametric pairwise (Bonferroni-adjusted)

Comparison	p-value (Dunn–Bonferroni)
AMI 20 mg/kg vs. Saline (Control)	< 0.001
AMI 20 mg/kg vs. VEN 10 mg/kg	0.00731
AMI 20 mg/kg vs. VEN 20 mg/kg	0.137
AMI 20 mg/kg vs. VEN 40 mg/kg	1
Saline (Control) vs. VEN 10 mg/kg	0.737
Saline (Control) vs. VEN 20 mg/kg	0.0693
Saline (Control) vs. VEN 40 mg/kg	< 0.001
VEN 10 mg/kg vs. VEN 20 mg/kg	1
VEN 10 mg/kg vs. VEN 40 mg/kg	0.0335
VEN 20 mg/kg vs. VEN 40 mg/kg	0.432

Experiment 3 — TST, VEN 20 mg/kg + codeine geometric series (n = 10 per group)

Tukey HSD — all pairwise comparisons

<table>
<tr><th>Comparison</th><th>Mean diff</th><th>Lower 95% CI</th><th>Upper 95% CI</th><th>p-value (Tukey)</th></tr>
<tr><td>AMI 20 mg/kg vs. Saline (Control)</td><td>+88.40</td><td>+31.54</td><td>+145.26</td><td>0.0004</td></tr>
<tr><td>AMI 20 mg/kg vs. VEN 20 + COD 10 mg/kg</td><td>+46.60</td><td>-10.26</td><td>+103.46</td><td>0.167</td></tr>
<tr><td>AMI 20 mg/kg vs. VEN 20 + COD 20 mg/kg</td><td>+47.80</td><td>-9.06</td><td>+104.66</td><td>0.147</td></tr>
<tr><td>AMI 20 mg/kg vs. VEN 20 + COD 40 mg/kg</td><td>+45.80</td><td>-11.06</td><td>+102.66</td><td>0.182</td></tr>
<tr><td>AMI 20 mg/kg vs. VEN 20 mg/kg</td><td>+28.50</td><td>-28.36</td><td>+85.36</td><td>0.678</td></tr>
<tr><td>Saline (Control) vs. VEN 20 + COD 10 mg/kg</td><td>-41.80</td><td>-98.66</td><td>+15.06</td><td>0.268</td></tr>
<tr><td>Saline (Control) vs. VEN 20 + COD 20 mg/kg</td><td>-40.60</td><td>-97.46</td><td>+16.26</td><td>0.298</td></tr>
<tr><td>Saline (Control) vs. VEN 20 + COD 40 mg/kg</td><td>-42.60</td><td>-99.46</td><td>+14.26</td><td>0.249</td></tr>
<tr><td>Saline (Control) vs. VEN 20 mg/kg</td><td>-59.90</td><td>-116.76</td><td>-3.04</td><td>0.0334</td></tr>
<tr><td>VEN 20 + COD 10 mg/kg vs. VEN 20 + COD 20 mg/kg</td><td>+1.20</td><td>-55.66</td><td>+58.06</td><td rowspan="2">1</td></tr>
<tr><td>VEN 20 + COD 10 mg/kg vs. VEN 20 + COD 40 mg/kg</td><td>-0.80</td><td>-57.66</td><td>+56.06</td></tr>
</table>

VEN 20 + COD 10 mg/kg vs. VEN 20 mg/kg	-18.10	-74.96	+38.76	0.934
VEN 20 + COD 20 mg/kg vs. VEN 20 + COD 40 mg/kg	-2.00	-58.86	+54.86	1
VEN 20 + COD 20 mg/kg vs. VEN 20 mg/kg	-19.30	-76.16	+37.56	0.915
VEN 20 + COD 40 mg/kg vs. VEN 20 mg/kg	-17.30	-74.16	+39.56	0.945

Dunnett's test — vs. Saline control

**Comparison**	**t-statistic**	**p-value (Dunnett)**
AMI 20 mg/kg vs. Saline (Control)	-4.593	0.000125
VEN 20 mg/kg vs. Saline (Control)	-3.112	0.0129
VEN 20 + COD 10 mg/kg vs. Saline (Control)	-2.172	0.128
VEN 20 + COD 20 mg/kg vs. Saline (Control)	-2.110	0.146
VEN 20 + COD 40 mg/kg vs. Saline (Control)	-2.213	0.118

Dunn's test — non-parametric pairwise (Bonferroni-adjusted)

<table>
<tr><th>Comparison</th><th>p-value
(Dunn–Bonferroni)</th></tr>
<tr><td>AMI 20 mg/kg vs. Saline (Control)</td><td>0.000608</td></tr>
<tr><td>AMI 20 mg/kg vs. VEN 20 + COD 10 mg/kg</td><td>0.171</td></tr>
<tr><td>AMI 20 mg/kg vs. VEN 20 + COD 20 mg/kg</td><td>0.12</td></tr>
<tr><td>AMI 20 mg/kg vs. VEN 20 + COD 40 mg/kg</td><td>0.168</td></tr>
<tr><td>AMI 20 mg/kg vs. VEN 20 mg/kg</td><td rowspan="4">1</td></tr>
<tr><td>Saline (Control) vs. VEN 20 + COD 10 mg/kg</td></tr>
<tr><td>Saline (Control) vs. VEN 20 + COD 20 mg/kg</td></tr>
<tr><td>Saline (Control) vs. VEN 20 + COD 40 mg/kg</td></tr>
<tr><td>Saline (Control) vs. VEN 20 mg/kg</td><td>0.272</td></tr>
<tr><td>VEN 20 + COD 10 mg/kg vs. VEN 20 + COD 20 mg/kg</td><td rowspan="6">1</td></tr>
<tr><td>VEN 20 + COD 10 mg/kg vs. VEN 20 + COD 40 mg/kg</td></tr>
<tr><td>VEN 20 + COD 10 mg/kg vs. VEN 20 mg/kg</td></tr>
<tr><td>VEN 20 + COD 20 mg/kg vs. VEN 20 + COD 40 mg/kg</td></tr>
<tr><td>VEN 20 + COD 20 mg/kg vs. VEN 20 mg/kg</td></tr>
<tr><td>VEN 20 + COD 40 mg/kg vs. VEN 20 mg/kg</td></tr>
</table>

Experiment 4 — TST, triple combination (n = 12 per group)

Tukey HSD — all pairwise comparisons

Comparison	Mean diff	Lower 95% CI	Upper 95% CI	p-value (Tukey)
AMI 20 mg/kg vs. COD 20 mg/kg	+58.00	-21.42	+137.42	0.278
AMI 20 mg/kg vs. Saline (Control)	+95.58	+16.16	+175.00	0.0095
AMI 20 mg/kg vs. norBNI 10 + VEN 20 + COD 20 mg/kg	+12.08	-67.34	+91.50	0.998
AMI 20 mg/kg vs. norBNI 10 + VEN 20 mg/kg	+32.75	-46.67	+112.17	0.83
AMI 20 mg/kg vs. norBNI 10 mg/kg	+81.50	+2.08	+160.92	0.041
COD 20 mg/kg vs. Saline (Control)	+37.58	-41.84	+117.00	0.733
COD 20 mg/kg vs. norBNI 10 + VEN 20 + COD 20 mg/kg	-45.92	-125.34	+33.50	0.539
COD 20 mg/kg vs. norBNI 10 + VEN 20 mg/kg	-25.25	-104.67	+54.17	0.936
COD 20 mg/kg vs. norBNI 10 mg/kg	+23.50	-55.92	+102.92	0.953
Saline (Control) vs. norBNI 10 + VEN 20 + COD 20 mg/kg	-83.50	-162.92	-4.08	0.0337
Saline (Control) vs. norBNI 10 + VEN 20 mg/kg	-62.83	-142.25	+16.59	0.2
Saline (Control) vs. norBNI 10 mg/kg	-14.08	-93.50	+65.34	0.995
norBNI 10 + VEN 20 + COD 20 mg/kg vs. norBNI 10 + VEN 20 mg/kg	+20.67	-58.75	+100.09	0.973
norBNI 10 + VEN 20 + COD 20 mg/kg vs. norBNI 10 mg/kg	+69.42	-10.00	+148.84	0.121
norBNI 10 + VEN 20 mg/kg vs. norBNI 10 mg/kg	+48.75	-30.67	+128.17	0.472

Dunnett's test — vs. Saline control

Comparison	t-statistic	p-value (Dunnett)
AMI 20 mg/kg vs. Saline (Control)	-3.532	0.00345
norBNI 10 mg/kg vs. Saline (Control)	-0.520	0.979
norBNI 10 + VEN 20 mg/kg vs. Saline (Control)	-2.322	0.0908

norBNI 10 + VEN 20 + COD 20 mg/kg vs. Saline (Control)	-3.086	0.0131
COD 20 mg/kg vs. Saline (Control)	-1.389	0.501

Dunn's test — non-parametric pairwise (Bonferroni-adjusted)

<table>
<tr><th>Comparison</th><th>p-value
(Dunn–Bonferroni)</th></tr>
<tr><td>AMI 20 mg/kg vs. COD 20 mg/kg</td><td>0.689</td></tr>
<tr><td>AMI 20 mg/kg vs. Saline (Control)</td><td>0.112</td></tr>
<tr><td>AMI 20 mg/kg vs. norBNI 10 + VEN 20 + COD 20 mg/kg</td><td rowspan="2">1</td></tr>
<tr><td>AMI 20 mg/kg vs. norBNI 10 + VEN 20 mg/kg</td></tr>
<tr><td>AMI 20 mg/kg vs. norBNI 10 mg/kg</td><td>0.00675</td></tr>
<tr><td>COD 20 mg/kg vs. Saline (Control)</td><td rowspan="4">1</td></tr>
<tr><td>COD 20 mg/kg vs. norBNI 10 + VEN 20 + COD 20 mg/kg</td></tr>
<tr><td>COD 20 mg/kg vs. norBNI 10 + VEN 20 mg/kg</td></tr>
<tr><td>COD 20 mg/kg vs. norBNI 10 mg/kg</td></tr>
<tr><td>Saline (Control) vs. norBNI 10 + VEN 20 + COD 20 mg/kg</td><td>0.287</td></tr>
<tr><td>Saline (Control) vs. norBNI 10 + VEN 20 mg/kg</td><td rowspan="3">1</td></tr>
<tr><td>Saline (Control) vs. norBNI 10 mg/kg</td></tr>
<tr><td>norBNI 10 + VEN 20 + COD 20 mg/kg vs. norBNI 10 + VEN 20 mg/kg</td></tr>
<tr><td>norBNI 10 + VEN 20 + COD 20 mg/kg vs. norBNI 10 mg/kg</td><td>0.0223</td></tr>
<tr><td>norBNI 10 + VEN 20 mg/kg vs. norBNI 10 mg/kg</td><td>0.255</td></tr>
</table>
