# Supplementary material for: Antidepressant-like Activity of a Triple Combination of Venlafaxine, Codeine and Nor-Binaltorphimine in the Mouse Tail Suspension Test
Source: Int J Mol Sci. 2026 Oct 29;27(21):9719. doi: 10.3390/ijms27219719 (PMC13763530; doi:10.3390/ijms27219719)
Supplement: Supplementary file 1 [file ijms-27-09719-s001.zip › Supplementary Table S3.pdf]

Supplementary Table S3. Pairwise effect sizes (Cohen's *d*, Hedges' *g*, Cliff's δ) for all comparisons

Table S3. Effect sizes for each treatment group compared to the saline control. Cohen's d uses the pooled SD; Hedges' g applies the small-sample bias correction factor (J = 1 – 3/(4n – 9)). Cliff's δ is a non-parametric effect-size index (range –1 to +1; 0 indicates complete overlap of the two distributions). Negative values denote a reduction in immobility (antidepressant-like effect) relative to saline control. Cohen's benchmark thresholds: |d| = 0.2 small, 0.5 medium, 0.8 large; |δ| = 0.15 small, 0.33 medium, 0.47 large.

Experiment 1 — FST, venlafaxine dose-response (n = 17 per group)

Group	Cohen's d	Hedges' g	Cliff's δ	% change vs Ctrl	Cohen interpretation
AMI 20 mg/kg	-4.03	-3.94	-1.00	-64.3%	extreme
VEN 10 mg/kg	-0.67	-0.65	-0.24	-14.9%	medium
VEN 20 mg/kg	-0.27	-0.27	-0.07	-4.3%	small
VEN 40 mg/kg	-0.60	-0.59	-0.20	-13.6%	medium

Experiment 2 — TST, venlafaxine dose-response (n = 12 per group)

Group	Cohen's d	Hedges' g	Cliff's δ	% change vs Ctrl	Cohen interpretation
AMI 20 mg/kg	-4.65	-4.49	-1.00	-71.5%	extreme
VEN 10 mg/kg	-1.14	-1.10	-0.64	-23.5%	large
VEN 20 mg/kg	-1.66	-1.60	-0.78	-36.5%	very large
VEN 40 mg/kg	-3.75	-3.62	-1.00	-66.2%	extreme

Experiment 3 — TST, VEN 20 mg/kg + codeine geometric series (n = 10 per group)

Group	Cohen's d	Hedges' g	Cliff's δ	% change vs Ctrl	Cohen interpretation
AMI 20 mg/kg	-1.76	-1.69	-0.82	-74.4%	very large
VEN 20 mg/kg	-1.28	-1.22	-0.68	-50.4%	
VEN 20 + COD 10 mg/kg	-0.80	-0.76	-0.38	-35.2%	medium
VEN 20 + COD 20 mg/kg	-0.89	-0.86	-0.52	-34.2%	large
VEN 20 + COD 40 mg/kg	-0.87	-0.83	-0.48	-35.9%	

Experiment 4 — TST, triple combination (n = 12 per group)

Group	Cohen's d	Hedges' g	Cliff's δ	% change vs Ctrl	Cohen interpretation
AMI 20 mg/kg	-1.25	-1.21	-0.57	-75.2%	very large
norBNI 10 mg/kg	-0.17	-0.17	+0.07	-11.1%	negligible
norBNI 10 + VEN 20 mg/kg	-0.71	-0.68	-0.34	-49.4%	medium
norBNI 10 + VEN 20 + COD 20 mg/kg	-1.06	-1.03	-0.53	-65.7%	large

COD 20 mg/kg	-0.40	-0.39	-0.15	-29.6%	small
