# Supplementary material for: Antidepressant-like Activity of a Triple Combination of Venlafaxine, Codeine and Nor-Binaltorphimine in the Mouse Tail Suspension Test
Source: Int J Mol Sci. 2026 Oct 29;27(21):9719. doi: 10.3390/ijms27219719 (PMC13763530; doi:10.3390/ijms27219719)
Supplement: Supplementary file 1 [file ijms-27-09719-s001.zip › Supplementary Table S4.pdf]

Supplementary Table S4. Complete docking matrix for the forty ligand–structure pairs across the eight human structures.

Ligand	Structure	Run 1	Run 2	Run 3	Mean ± SD	Anchor distance, top mode (Å)	Anchor-consistent mode rank	Anchor-consistent score	Anchor-consistent distance (Å)	LE
Venlafaxine	SERT (5I6X)	-7.93	-7.93	-7.92	-7.93 ± 0.00	3.86	1	-7.95	3.86	0.397
Venlafaxine	NET (8Y93)	-8.26	-8.25	-8.25	-8.26 ± 0.01	4.80	6	-7.70	3.62	0.385
Venlafaxine	DAT (9EO4)	-8.69	-8.69	-8.70	-8.69 ± 0.01	4.49	2	-8.55	3.77	0.428
Venlafaxine	μ-OR (active) (8EFO)	-6.95	-6.96	-7.01	-6.97 ± 0.03	3.06	1	-6.97	3.06	0.349
Venlafaxine	μ-OR (inactive) (9PXU)	-6.85	-6.93	-6.83	-6.87 ± 0.05	8.44	none	–	–	–
Venlafaxine	κ-OR (inactive) (4DJH)	-6.97	-7.03	-6.96	-6.99 ± 0.04	3.03	1	-6.97	3.03	0.349
Venlafaxine	κ-OR (G_i-coupled) (8VVE)	-6.99	-6.98	-7.03	-7.00 ± 0.02	11.34	17	-5.62	3.16	0.281
Venlafaxine	δ-OR (4N6H)	-7.29	-6.89	-7.35	-7.17 ± 0.25	5.13	4	-6.94	3.08	0.347
Amitriptyline	SERT (5I6X)	-7.17	-7.17	-7.23	-7.19 ± 0.03	3.26	1	-7.17	3.26	0.342
Amitriptyline	NET (8Y93)	-8.77	-8.80	-8.79	-8.79 ± 0.02	3.69	1	-8.84	3.69	0.421
Amitriptyline	DAT (9EO4)	-7.61	-7.62	-7.61	-7.61 ± 0.00	7.20	11	-6.33	3.54	0.301
Amitriptyline	μ-OR (active) (8EFO)	-6.40	-6.87	-6.86	-6.71 ± 0.27	5.25	4	-6.22	2.98	0.296
Amitriptyline	μ-OR (inactive) (9PXU)	-6.59	-6.34	-6.58	-6.50 ± 0.14	4.59	none	–	–	–
Amitriptyline	κ-OR (inactive) (4DJH)	-6.05	-6.04	-6.16	-6.08 ± 0.06	4.46	2	-5.87	3.95	0.280

Amitriptyline	κ-OR (G_i-coupled) (8VVE)	-5.92	-6.06	-5.68	-5.89 ± 0.19	15.39	3	-5.54	3.94	0.264
Amitriptyline	δ-OR (4N6H)	-6.28	-6.14	-6.05	-6.16 ± 0.11	8.03	3	-5.82	3.54	0.277
Codeine	SERT (5I6X)	-8.25	-8.25	-8.23	-8.25 ± 0.01	4.87	17	-7.01	3.82	0.318
Codeine	NET (8Y93)	-9.79	-9.77	-9.75	-9.77 ± 0.02	4.20	3	-7.86	3.81	0.357
Codeine	DAT (9EO4)	-7.52	-7.52	-7.52	-7.52 ± 0.00	4.86	9	-6.24	3.92	0.284
Codeine	μ-OR (active) (8EFO)	-8.00	-8.00	-7.99	-8.00 ± 0.01	5.38	2	-7.68	3.59	0.349
Codeine	μ-OR (inactive) (9PXU)	-7.87	-7.81	-7.79	-7.82 ± 0.04	5.30	none	–	–	–
Codeine	κ-OR (inactive) (4DJH)	-7.48	-7.50	-7.48	-7.49 ± 0.02	10.77	7	-6.70	3.94	0.304
Codeine	κ-OR (G_i-coupled) (8VVE)	-7.10	-7.10	-7.14	-7.12 ± 0.03	7.51	19	-5.89	3.35	0.268
Codeine	δ-OR (4N6H)	-7.16	-7.15	-7.16	-7.16 ± 0.01	3.43	1	-7.15	3.43	0.325
Morphine	SERT (5I6X)	-9.06	-9.03	-9.04	-9.04 ± 0.02	6.59	none	–	–	–
Morphine	NET (8Y93)	-9.66	-9.70	-9.71	-9.69 ± 0.02	4.24	4	-7.65	3.79	0.364
Morphine	DAT (9EO4)	-7.75	-7.76	-7.75	-7.75 ± 0.00	4.89	2	-7.36	3.99	0.351
Morphine	μ-OR (active) (8EFO)	-8.02	-8.00	-8.02	-8.01 ± 0.01	3.10	1	-7.99	3.10	0.381
Morphine	μ-OR (inactive) (9PXU)	-7.96	-7.97	-7.96	-7.96 ± 0.01	8.92	none	–	–	–
Morphine	κ-OR (inactive) (4DJH)	-7.28	-7.29	-7.29	-7.29 ± 0.01	10.76	8	-6.69	3.97	0.318

Morphine	κ-OR (G_i-coupled) (8VVE)	-7.06	-7.06	-7.04	-7.05 ± 0.01	7.78	none	–	–	–
Morphine	δ-OR (4N6H)	-7.09	-7.09	-7.10	-7.09 ± 0.00	6.57	2	-7.06	3.53	0.336
nor-Binaltorphimine	SERT (5I6X)	-3.65	-3.51	-3.31	-3.49 ± 0.17	5.46	none	–	–	–
nor-Binaltorphimine	NET (8Y93)	-4.45	-3.54	-4.49	-4.16 ± 0.54	6.63	none	–	–	–
nor-Binaltorphimine	DAT (9EO4)	-8.61	-8.34	-8.58	-8.51 ± 0.15	4.69	none	–	–	–
nor-Binaltorphimine	μ-OR (active) (8EFO)	-10.62	-10.61	-10.54	-10.59 ± 0.04	4.72	3	-10.14	3.80	0.207
nor-Binaltorphimine	μ-OR (inactive) (9PXU)	-10.35	-10.36	-10.37	-10.36 ± 0.01	6.11	none	–	–	–
nor-Binaltorphimine	κ-OR (inactive) (4DJH)	-9.94	-9.94	-9.92	-9.93 ± 0.01	3.00	1	-9.89	3.00	0.202
nor-Binaltorphimine	κ-OR (G_i-coupled) (8VVE)	-10.23	-10.21	-10.22	-10.22 ± 0.01	3.26	1	-10.20	3.26	0.208
nor-Binaltorphimine	δ-OR (4N6H)	-10.01	-9.94	-9.81	-9.92 ± 0.10	2.95	1	-9.98	2.95	0.204

Affinities in kcal mol^{-1}. Runs 1 to 3 are the top-ranked mode of three independent searches with seeds 20240301, 20240302 and 20240303. The anchor-consistent mode is the best-scoring mode, among the twenty retained in the reference run, in which a cationic ligand nitrogen lies within 4.0 Å of a carboxylate oxygen of the conserved aspartate of transmembrane helix 3; its rank in the score-ordered list is given. "none" indicates that no such mode was present. In the inactive μ-opioid receptor (9PXU) the deposited naloxone itself lies 5.71 Å from Asp149, so the criterion is not informative for that structure. LE, ligand efficiency, the negative of the anchor-consistent score divided by the number of heavy atoms. The Vina score is an empirical ranking function, not a thermodynamic binding free energy, and scales with molecular size.
