# Supplementary material for: Antidepressant-like Activity of a Triple Combination of Venlafaxine, Codeine and Nor-Binaltorphimine in the Mouse Tail Suspension Test
Source: Int J Mol Sci. 2026 Oct 29;27(21):9719. doi: 10.3390/ijms27219719 (PMC13763530; doi:10.3390/ijms27219719)
Supplement: Supplementary file 1 [file ijms-27-09719-s001.zip › Supplementary Table S5.pdf]

Supplementary Table S5. Redocking validation of the eight human experimental structures and definition of the search box.

Structure	PDB ID	Method, resolution	Source organism (UniProt)	Co-crystallised ligand (CCD code)	RMSD (Å)	Score (kcal mol^{-1})	Crystal anchor distance (Å)	Anchor residue	Box centre x, y, z (Å)	Box size (Å)
SERT	5I6X	X-ray, 3.14 Å	Homo sapiens (P31645)	Paroxetine (8PR)	0.65	-10.78	3.09	Asp98	-32.61, -21.39, 1.85	22 × 22 × 22
NET	8Y93	cryo-EM, 3.00 Å	Homo sapiens (P23975)	Amitriptyline (TP0)	0.88	-9.33	4.46	Asp75	117.55, 115.15, 96.92	22 × 22 × 22
DAT	9EO4	cryo-EM, 2.66 Å	Homo sapiens (Q01959)	Cocaine (COC)	0.60	-8.96	3.27	Asp79	130.28, 125.47, 126.46	22 × 22 × 22
μ-OR (active)	8EFO	cryo-EM, 2.80 Å	Homo sapiens (P35372)	PZM21 (8QY)	1.39	-8.17	3.22	Asp149$^{3.32}$	185.52, 171.44, 172.73	22 × 22 × 22
μ-OR (inactive)	9PXU	cryo-EM, 3.40 Å	Homo sapiens (P35372)	Naloxone (A1APV)	1.04	-8.53	5.71	Asp149$^{3.32}$	156.50, 146.79, 106.16	22 × 22 × 22
κ-OR (inactive)	4DJH	X-ray, 2.90 Å	Homo sapiens (P41145)	JDTic (JDC)	0.42	-10.78	2.62	Asp138$^{3.32}$	3.68, -24.01, 59.47	22 × 22 × 22
κ-OR (G_i-coupled)	8VVE	cryo-EM, 3.30 Å	Homo sapiens (P41145)	nor-Binaltorphimine (A1AD6)	0.82	-10.39	3.40	Asp138$^{3.32}$	166.73, 158.65, 126.95	22 × 22 × 26
δ-OR	4N6H	X-ray, 1.80 Å	Homo sapiens (P41143)	Naltrindole (EJ4)	0.17	-11.51	2.87	Asp128$^{3.32}$	-5.91, -69.37, 70.81	22 × 22 × 22

RMSD, symmetry-corrected heavy-atom root-mean-square deviation of the top-ranked mode from the deposited pose, computed in the experimental frame without superposition and minimised over all graph automorphisms of the ligand. All eight structures met the 2.0 Å criterion set a priori. The crystal anchor distance is the shortest separation, in the deposited structure, between a ligand nitrogen and a carboxylate oxygen of the conserved aspartate of transmembrane helix 3; it defines the 4.0 Å criterion applied to the docked poses, which the noradrenaline transporter and the inactive μ-opioid receptor do not themselves satisfy. Superscripts give Ballesteros–Weinstein numbering.
